# Mutations in *Hnrnpa1* cause congenital heart defects

**DOI:** 10.1172/jci.insight.98555

**Published:** 2018-01-25

**Authors:** Zhe Yu, Paul L.F. Tang, Jing Wang, Suying Bao, Joseph T. Shieh, Alan W.L. Leung, Zhao Zhang, Fei Gao, Sandra Y.Y. Wong, Andy L.C. Hui, Yuan Gao, Nelson Dung, Zhi-Gang Zhang, Yanhui Fan, Xueya Zhou, Yalun Zhang, Dana S.M. Wong, Pak C. Sham, Abid Azhar, Pui-Yan Kwok, Patrick P.L. Tam, Qizhou Lian, Kathryn S.E. Cheah, Binbin Wang, You-Qiang Song

**Affiliations:** 1 School of Biomedical Sciences, Joint Laboratories of Matrix Biology and Diseases, The University of Hong Kong, Hong Kong, China.; 2 National Research Institute for Family Planning, Beijing, China.; 3 Institute for Human Genetics and Department of Pediatrics, School of Medicine, University of California San Francisco, San Francisco, California, USA.; 4 Department of Medicine and Ophthalmology,; 5 Department of Psychiatry,; 6 Centre for Genome Sciences, and; 7 State Key Laboratory for Cognitive and Brain Sciences, The University of Hong Kong, Hong Kong, China.; 8 Institute of Biotechnology & Genetic Engineering, University of Karachi, Karachi, Pakistan.; 9 Cardiovascular Research Institute, School of Medicine, University of California San Francisco, San Francisco, California, USA.; 10 Embryology Unit, Children’s Medical Research Institute, School of Medical Sciences, University of Sydney, Westmead, New South Wales, Australia.; 11 The University of Hong Kong Shenzhen Institute of Research and Innovation and; 12 The University of Hong Kong-Southern University of Science and Technology Joint Laboratories of Matrix Biology and Diseases, The University of Hong Kong, Hong Kong, China.

**Keywords:** Development, Genetics, Cardiovascular disease, Heart failure, Organogenesis

## Abstract

Incomplete penetrance of congenital heart defects (CHDs) was observed in a mouse model. We hypothesized that the contribution of a major genetic locus modulates the manifestation of the CHDs. After genome-wide linkage mapping, fine mapping, and high-throughput targeted sequencing, a recessive frameshift mutation of the heterogeneous nuclear ribonucleoprotein A1 (*Hnrnpa1*) gene was confirmed (*Hnrnpa1^ct^*). *Hnrnpa1* was expressed in both the first heart field (FHF) and second heart field (SHF) at the cardiac crescent stage but was only maintained in SHF progenitors after heart tube formation. *Hnrnpa1^ct/ct^* homozygous mutants displayed complete CHD penetrance, including truncated and incomplete looped heart tube at E9.5, ventricular septal defect (VSD) and persistent truncus arteriosus (PTA) at E13.5, and VSD and double outlet right ventricle at P0. Impaired development of the dorsal mesocardium and sinoatrial node progenitors was also observed. Loss of *Hnrnpa1* expression leads to dysregulation of cardiac transcription networks and multiple signaling pathways, including BMP, FGF, and Notch in the SHF. Finally, two rare heterozygous mutations of *HNRNPA1* were detected in human CHDs. These findings suggest a role of *Hnrnpa1* in embryonic heart development in mice and humans.

## Abstract 2

Heterogeneous nuclear ribonucleoprotein A1 (Hnrnpa1) is essential for embryonic heart development in both mice and humans.

## Introduction

In humans, nearly 1% of newborns are affected by congenital heart defects (CHDs), and epidemiological studies have suggested significant genetic contribution to the manifestation of CHDs (1–3). While most CHD cases occur sporadically, some familial cases have shown Mendelian inheritance patterns (4). Although the more common types of CHDs are complex disorders, mutations in single genes, such as *NKX2-5*, *GATA4*, and *TBX1*, were found to associate with rare forms of CHDs (1, 5–7).

Collagens are major components of the extracellular matrix; they play a key role in the induction of cell differentiation and tissue morphogenesis. We elucidated the function of IIA in *IIA* procollagen-deficient mice, generated by deleting exon II of *Col2a1* in a hybrid genetic background (8). However, mice homozygous for the *IIA*-null mutation (*IIA^−/−^*) displayed a variable severity of the congenital malformations, ranging from lethal heart malformation and craniofacial defects to near-normal phenotypes (Supplemental Figure 1; supplemental material available online with this article; https://doi.org/10.1172/jci.insight.98555DS1). To assess the heart defect phenotypes with different genetic backgrounds, three heterozygous *IIA*^+/−^ mutant mouse lines enriched with C57BL/6N, 129/SvEv, and ICR background were established, and the inheritance of the CHD/death phenotype was evaluated by the Mendelian ratio of the *IIA* genotypes in *IIA^+/−^* × *IIA^+/−^* crosses of different genetic backgrounds. However, among the *IIA*^+/−^ mutant mouse lines, only the *IIA*-null population from C57-*IIA^+/−^* × C57-*IIA^+/−^* crosses was significantly underrepresented, whereas ICR-*IIA^+/−^* × C57-*IIA^+/−^* crosses produced a normal Mendelian ratio (Supplemental Table 1). Thus, we hypothesized that the incomplete penetrance of CHD phenotypes was due to the involvement of an additional CHD-causing genetic factor rather than a random shift of the phenotypes. The CHD-causing genetic locus might be a mutant gene that was created during the targeted disruption of *Col2a1* and was only successfully fixed in the congenic C57-*IIA^+/−^* mouse line. The suspected genetic lethality may therefore be inherited in a recessive manner and linked with the mutant *Col2a1* allele, as the locus was nonpathogenic in C57-*IIA^+/−^* heterozygotes. The *IIA^−/−^* genotype alone was not sufficient for the manifestation of CHD phenotypes.

The CHD candidate locus was then mapped on chromosome 15qF3, which is approximately 9 cM away from the *Col2a1* locus. The cardiac phenotype of this particular line was lost after crossing over with the allele from C57BL/6N, 129/SvEv, or ICR, further supporting a recessive inheritance model. Fine mapping using a congenic set of C57-*IIA^−/−^* embryos narrowed down the candidate region to 1.2 Mb (chr15 from 102.29–103.49 Mb) and confirmed a recessive Mendelian inheritance pattern for this new genetic locus. A frameshift CT deletion in exon 5 of the heterogeneous nuclear ribonucleoprotein A1 (*Hnrnpa1*) gene was detected by targeted next-generation sequencing (NGS) of the candidate region and confirmed by Sanger sequencing (we refer to the mutant allele as *Hnrnpa1^ct^*). Further experiments demonstrate that the *Hnrnpa1^ct/ct^* mutation alone is sufficient to cause heart defects at different developmental stages, including E9.5, E13.5, and at birth (Table 1).

hnRNP A1, a member of the hnRNP A/B subfamily, is reported to take part in pre-mRNA alternative splicing, mRNA export and turnover, translation, miRNA processing, and the regulation of telomeres (9–13). A recent study revealed that, during smooth muscle differentiation, hnRNP A1 directly binds to the promoters of *MEF2c*, *SRF*, and myocardin (*Myocd*) to activate their transcription (14). MEF2c is a transcription factor required for proper outflow tract (OFT) alignment, regulation of anterior heart field (AHF), and cardiomyocyte differentiation (15); it is associated with congenital OFT defects in human patients (16). Myocardin forms a complex with SRF to activate the expression of downstream cardiac genes (17). In vitro experiments show that hnRNP A1 promotes the alternative translation of FGF2 at 4 IRES-dependent initiation sites (18). Isoform-specific ablation of FGF2 in mice has been shown to cause different heart defects in a sex-dependent manner (19). Previous reports have proposed that FGF2 may drive specification of cardiogenic lineage in the anterior lateral plate mesoderm (ALPM) (20, 21), promote proliferation of cardiomyocytes and vascular formation (22), and control the differentiation of resident cardiac precursors into cardiomyocytes in neonatal hearts (23). According to these results, we propose that *Hnrnpa1* plays an essential role during cardiac development, and we are the first to our knowledge to demonstrate that mutations in *Hnrnpa1* cause heart defects during early cardiac developmental stages.

## Results

### Identification of Hnrnpa1 as an independent disease gene during cardiac development.

**Figure 1:**
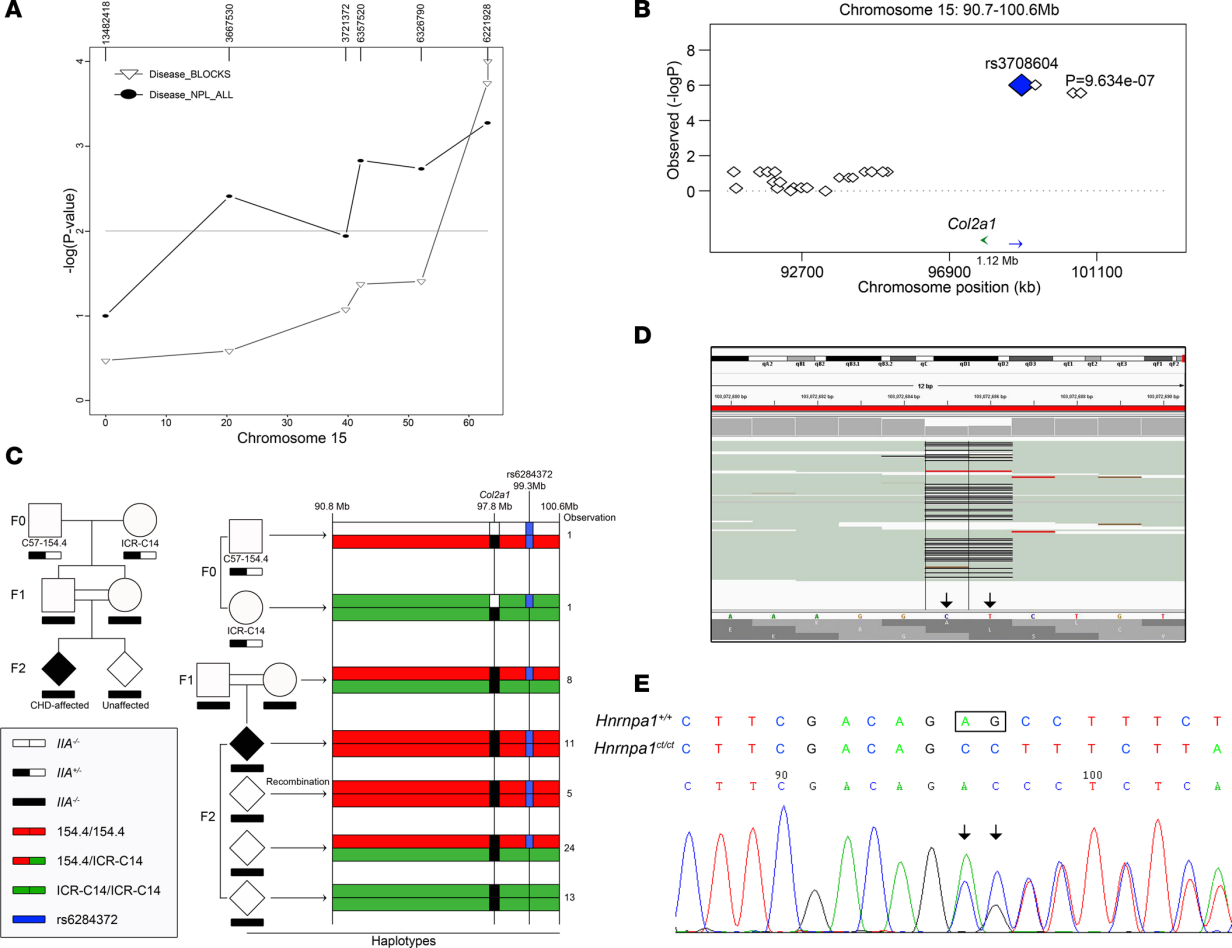
Identification of a de novo CT deletion in *Hnrnpa1*. (**A**) Chromosome 15–linked congenital heart defect (CHD) locus. The *x* axis represents the relative location of markers on a chromosome, and the *y* axis represents the –log(*P*) for nonparametric linkage–ALL (NPL-ALL) scores and BLOCK recessive scores. (**B**) Transmission disequilibrium test (TDT) for the combined data set from E9.5 and P0. We genotyped 40 more SNP markers for the region of chr15:90.7–100.6 Mb. Significant association with the heart defect phenotype was found starting from chr15:98.95 Mb, consistent with the NPL analysis in the embryonic lethality data set. We hypothesized the new CHD locus should be located beyond 98.95 Mb, where alleles from the C57BL/6N mutant were first shown to be predominately transmitted to affected individuals. We defined this segment, which independently assorted with the IIA genotype, as a breakpoint between *Col2a1* and the new CHD locus. The breakpoint was shown to be flanked by markers rs8277842 at 98.6 Mb and rs3708604 at 98.95 Mb on chromosome 15. The green arrow pointing to the left indicates the position of *Col2a1* and the blue arrow pointing to the right indicates the breakpoint. For both **A** and **B**, *P* < 0.001 was considered as significant. (**C**) Haplotype analysis in affected litters. The same region of the TDT analysis presented in chr15:90.8–100.6 Mb is shown. The haplotype information (154.4) is mainly inferred from rs6284372. In this pedigree, there are 8 F1, all bearing a heterozygous configuration for this segment. F2 collected from intercrossing F1 provide 3 main haplotypes: 154.4/154.4, 154.4/ICR-C14, and ICR-C14/ICR-C14. Among 53 individuals in F2, all 11 affected individuals was found to be carrying the 154.4/154.4 haplotype; while the unaffected individuals have a distribution of 5:24:13 for the 3 mentioned haplotype configurations, respectively. Markers do not appear to segregate in chr15:90.8–98.6 Mb, probably due to our selection of the *IIA*-null allele for a procollagen IIA–deficient mutant mouse line. Therefore, the haplotype for this region was inferred with an assumption that no recombination happened in the region with the pedigree under study. (**D**) Targeted sequencing of the 1.2-Mb candidate region detected a 2-base deletion in *Hnrnpa1* gene. A screen shot from the IGV browser shows the deletion of CT in *Hrnrnpa1* in a heterozygous mouse. (**E**) Sanger sequencing confirmed the presence of CT deletion in *Hnrnpa1*.

A de novo mutation was generated during the targeted disruption of exon 2 of the *Col2a1* gene. We hypothesized that a CHD-causing genetic locus already existed in the *IIA*-null mouse line with hybrid genetic background and generated a mapping panel for genome-wide linkage analysis in the F2 generation of mice from the C57-*IIA^+/−^* × ICR-*IIA^+/−^* crosses. A total of 117 SNPs with alleles differentiating C57BL/6J from ICR at a density of 20 cM were selected from the genome. Seventy-two P0 newborns from 8 litters with 11 CHD cases and thirty-eight E9.5 embryos from 3 litters with 6 CHD cases were genotyped. Using Simwalk2, which contains BLOCK statistics and is the most powerful method to detect linkage in a recessive model, a significant linkage on chromosome 15 at 60 cM was detected with –log(*P*) = 4 in nonparametric linkage (NPL) ALL statistics and –log(*P*) > 3 in BLOCK statistics in the recessive model (Figure 1A and Supplemental Figures 2 and 3). With increased marker density in this region, the data sets showed a genome-wide significant NPL, with –log(*P*) = 4 in the NPL ALL score for both E9.5 and P0. Moreover, the NPL result suggests that the defects in the two developmental stages were possibly due to the same genetic variant and, thus, originated from the same developmental process. The data sets of the two stages were combined to perform transmission disequilibrium test (TDT) analysis using 40 markers. Significant association (*P* = 9.634 × 10^–7^) with the phenotype was found starting from 98.95 Mb (Figure 1B). We hypothesized that the genetic locus for CHD is located beyond 98.95 Mb toward the end of chromosome 15, where the *IIA*-null alleles from C57-*IIA^+/−^* mice are predominantly transmitted to affected individuals. To better understand the transmission pattern of the segment beyond the breakpoint, we traced the transmission of the haplotypes in the affected litters in a subset of the mapping pedigrees. To avoid the phasing problem, we selected 18 SNPs that were homozygous for one of the F0 parents but heterozygous for another (Supplemental Table 2). The C57-*IIA^+/−^* mutant mouse line was found to be very heterogeneous in this region. Assuming the CHD-causing haplotype (shown in red in Figure 1C) (referred to herein as 154.4) is linked to the *IIA* locus, the white bar (as shown in Figure 1C) should be inherited from C57BL/6N, acquired from backcrossing. The ICR-*IIA^+/−^* line, on the other hand, is very homogenous (green bar, Figure 1C). There is one SNP (rs6284372) at 99.3Mb, however, that was found to be homozygous in the 154.4 haplotype but heterozygous in ICR. The haplotype information is mainly inferred from this SNP. The 154.4/154.4 homozygous genotype was predominantly transmitted to affected individuals (Figure 1C). Given that some of the unaffected mutants also carried the same haplotype, the causative mutation was probably more distant and estimated to be approximately 9 cM away from the region, which spanned across *Col2a1* and was inherited from the C57-*IIA^+/−^* mutant mouse line. Consistent with our hypothesis, this haplotype configuration did not match with that of our C57BL/6N wild-type stock or C57BL/6J in the NCBI database. The configuration matched that of our 129/SvEv wild-type stock and 129S1/SvImJ from the Mouse Phenome database (Supplemental Figure 4). Since we did not observe any cardiac abnormalities in the mice with genotype of 129-*IIA^−/−^* in embryonic or postnatal stages (Supplemental Table 1), and assuming that the ES cell used for generating the *IIA*-null mutant was indeed a hybrid of 129S1/SvImJ and 129X1/SvJ, we hypothesized that this segment of DNA contained a mutation that caused CHD, which was generated during the targeted disruption of *Col2a1* and cosegregated with the *IIA*-null locus. This genetic locus was fixed in the C57-*IIA^+/−^* mutant mouse line during backcrosses.

### Establishment of an IIA wild-type CHD mouse line by identifying a recombinant of Col2a1 and rs3218302 loci.

To further define whether the *IIA*-null allele contributed to the occurrence of CHD phenotype, we generated more C57-*IIA^−/−^* embryos and conducted fine mapping using additional SNPs. Supplemental Figure 1G shows the prevalence of CHD in each litter collected from C57-*IIA^+/−^* × C57-*IIA^+/−^* crosses We genotyped 8 affected and 8 unaffected C57-*IIA^−/−^* embryos from the 6 litters collected in the previous analysis. The 6 litters consisted of 2 litters with 100% penetrance for CHD, 2 litters with 0%, and 2 litters with 50%, providing us with a comprehensive and representative set of observations. The embryos were genotyped for 18 markers of the original mapping panel, and rs32183020, which is approximately 9 cM away from the *Col2a1* locus, was used to mark the end of the candidate region. Their genotype configurations are summarized in Figure 1C. Two important features were noted. First, *Col2a1* was indeed flanked by 129S1/SvImJ genetic material. Second, rs32183020 was able to differentiate the affected and unaffected CHD C57-*IIA^−/−^* mutants, where all CHD C57-*IIA^−/−^* mutants harbored a homozygous “CC” genotype of rs32183020, and all non-CHD C57-*IIA^−/−^* mutants carried at least one copy of the “T” allele of rs32183020 from C57BL/6J. This result not only provided independent confirmation of our linkage analysis, but also explained why the incomplete penetrance was previously observed in C57-*IIA*^−/−^ mice. Finally, we narrowed down the candidate region to 1.2 Mb, with 43 transcripts encoded by 34 genes and 4 miRNAs.

With the identification of the “C” allele of rs32183020, which was strongly associated with the CHD mutant allele, we selected a recombinant mouse carrying the wild-type genotype of *IIA* and a heterozygous “CT” at rs32183020 from the C57-*IIA^+/−^* × C57-*IIA^+/−^* crosses. The selected recombinant mouse was crossed back to the C57BL/6N wild-type mouse to establish a mouse line that only contained the CHD mutant allele on chromosome 15 of the wild-type *IIA*.

### A frameshift mutation of the Hnrnpa1 gene causes the CHD phenotype.

We performed high-throughput targeted sequencing to identify a variant associated with CHD. The heterozygous (CT genotype) rs32183020 mouse was selected, and DNA was isolated for sequencing. Custom probes (Roche NimbleGen) were designed to target the 1.2-Mb candidate locus (chr15: 102.29–103.49 Mb) and were selected using the NimbleGen 2.1 M-probe; the captured fragments were sequenced at an average of 97 times with paired-end 90-bp reads by using NGS. We generated a sequence length of 184.4 Mb with 1.4% duplication rate. 96% of the target region was covered.

We used SAMtools and default filtering parameters to obtain the variants. Twenty-three SNPs and twenty-eight indels were detected compared with the reference, of which eight SNPs and sixteen indels were also observed in dbSNP128. Among the remaining variants, the only exonic variant led to a confidently predicted loss of function of *Hnrnpa1*, and a 2-base deletion (i.e., c.539_540del; p.180_180del, NM_001039129 in *Hnrnpa1*) was annotated by both RefSeq and UCSC gene annotation (Figure 1D). Moreover, we confirmed the same variants by Sanger sequencing (Figure 1E).

### Hnrnpa1^ct/ct^ mutant mice display complete penetrance of CHDs at multiple cardiac developmental stages.

**Figure 2:**
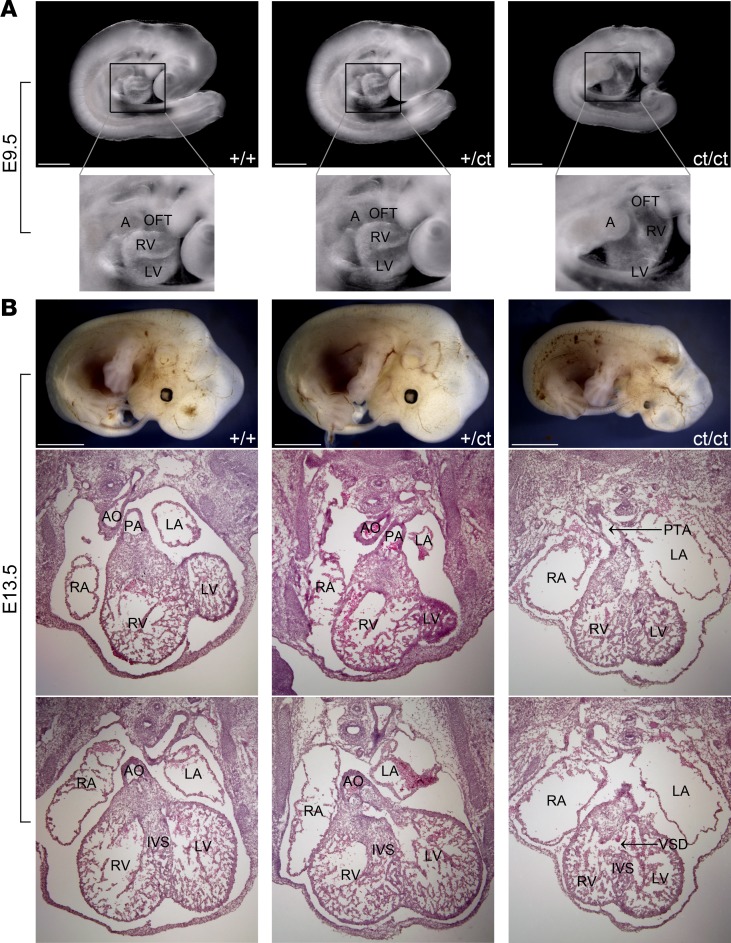
*Hnrnpa1^ct/ct^* mutant mice display severe congenital cardiac malformations at E9.5 and E13.5. Hearts from 5 wild-type littermate controls, 5 *Hnrnpa1^+/ct^* heterozygous mutants, and 3 *Hnrnpa1^ct/ct^* homozygous mutants were sectioned and stained with hematoxylin and eosin. (**A**) A truncated and incomplete looped heart tube in *Hnrnpa1^ct/ct^* homozygous mutants at E9.5. Scale bar: 500 μm. Original magnification, ×5.1. (**B**) The E13.5 *Hnrnpa1^ct/ct^* homozygous mutant heart with PTA and VSD. RA, right atrium; LA, left atrium; RV, right ventricle; LV, left ventricle; AO, aorta; PA, pulmonary artery; IVS, interventricular septum; PTA, persistent truncus arteriosus; VSD, ventricular septal defect. Scale bar: 2 mm.

To further confirm the suspected role of *Hnrnpa1* in the pathogenesis of CHDs, we used the *Hnrnpa1^+/ct^* heterozygotes for crosses. In the following text, *IIA^+/+^* is included in all genotypes. We collected 79 *Hnrnpa1^ct/ct^,* 208 *Hnrnpa1^+/ct^,* and 113 *Hnrnpa1^+/+^* mice at different developmental stages (Table 1). At E9.5, the distribution of 3 genotypes was consistent with Mendelian law. In *Hnrnpa1^+/ct^* heterozygotes, heart tube morphology was normal at E9.5, similar to that of wild-type littermate controls (Figure 2A). However, truncated and incomplete looped heart tubes with hypomorphic OFTs and right ventricles were only observed in *Hnrnpa1^ct/ct^* homozygotes (Fisher’s exact test: *P* = 4.7 × 10^–68^; Figure 2A). Three *Hnrnpa1^ct/ct^* homozygotes were also collected at E13.5, with marked retarded growth. Histological analysis revealed normal anatomy in both wild-type littermate controls and *Hnrnpa1^+/ct^* heterozygotes, whereas all 3 *Hnrnpa1^ct/ct^* homozygotes displayed ventricular septal defect (VSD) and persistent truncus arteriosus (PTA), which was caused by complete failure of OFT septation (Figure 2B). At P0, a substantial underrepresentation of the *Hnrnpa1^ct/ct^* homozygotes was observed, indicating embryonic lethality. The ratio between wild-type and heterozygous mutant P0 mice approximated the expected value according to Mendelian law. The P0 distribution is similar as that in a recent report (24). The two P0 *Hnrnpa1^ct/ct^* homozygotes displayed smaller body size and truncated head, compared with wild-type littermate controls and *Hnrnpa1^+/ct^* heterozygotes (Supplemental Figure 5). According to optical projection tomography (OPT) analyses, VSD was observed in one *Hnrnpa1^ct/ct^* homozygote, while double outlet right ventricle (DORV) was detected in another one (Supplemental Figure 5). CHDs were also only observed in *Hnrnpa1^ct/ct^* homozygous mutant newborns (Fisher’s exact test: *P* = 2.2 × 10^–4^). All these results demonstrate the *Hnrnpa1^ct/ct^* homozygous mutation alone is sufficient to cause CHDs in our mouse model.

### Hnrnpa1 is expressed in both the first heart field and second heart field cardiac lineages at the cardiac crescent stage but is maintained only in second heart field progenitors after heart tube formation.

**Figure 3:**
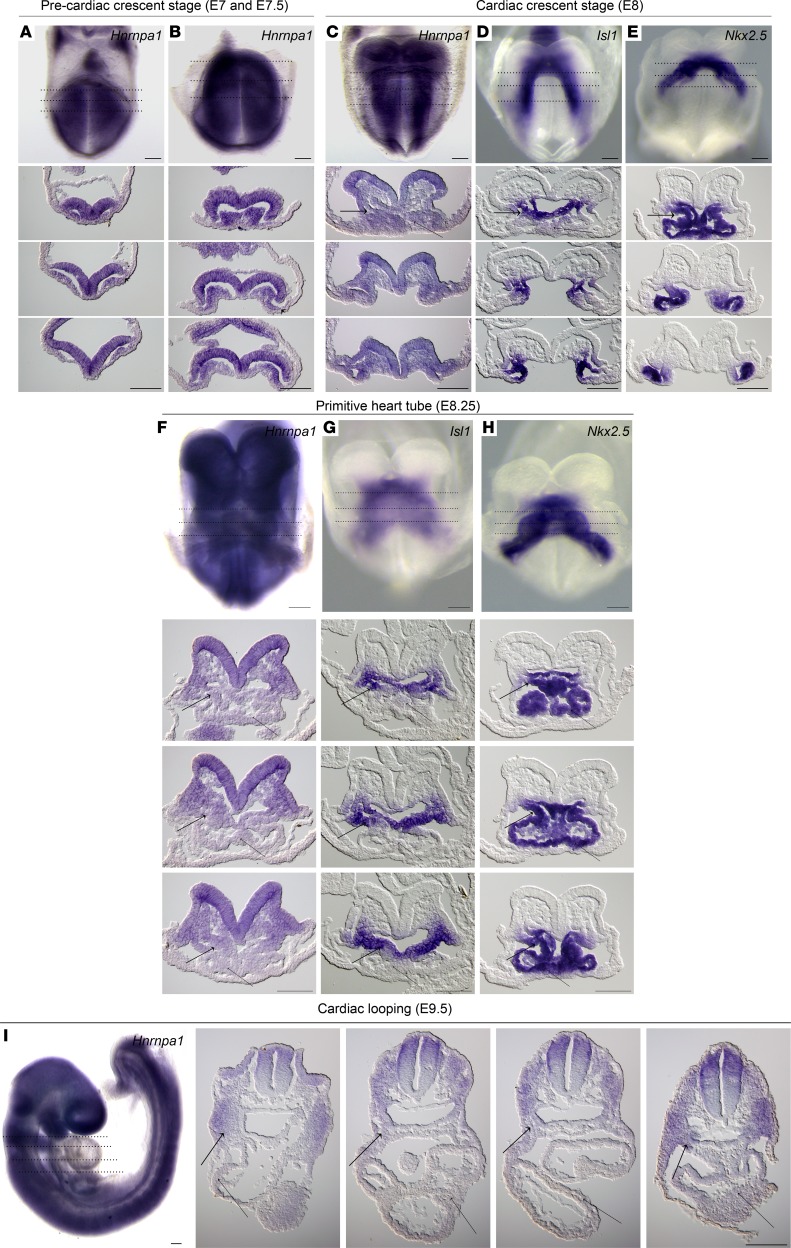
Whole-mount in situ analysis of wild-type embryos stained for *Hnrnpa1* mRNA at different embryonic days. Wild-type embryos were stained for *Hnrnpa1* mRNA, and corresponding sections are shown from arterial pole to venous pole. At E8 and E8.25, staining for *Isl1* and *Nkx2.5* was also performed. *Isl1* labels SHF cardiac progenitors, whereas *Nkx2.5* labels both the FHF and SHF lineages. At each stage, results from 1 of 3 representative experiments are displayed. (**A**) E7; (**B**) E7.5; (**C–E**) E8 (2 somite pairs); (**F–H**) E8.25 (6 somite pairs); and (**I**) E9.5 (20 somite pairs). (**A** and **B**) Expression of *Hnrnpa1* in the pre–cardiac crescent stages. *Hnrnpa1* mRNA is detected in the ALPM (short arrow). (**C**) During the cardiac crescent stage, *Hnrnpa1* mRNA is expressed in both differentiated myocardium (thin long arrow) and splanchnic mesoderm (wide long arrow). (**F**) After the formation of primitive heart tube, *Hnrnpa1* mRNA maintains its strong expression in the splanchnic mesoderm but with much lower expression level in the differentiated myocardium. (**I**) In E9.5 wild-type embryo, *Hnrnpa1* mRNA continues to be strongly expressed in the splanchnic mesoderm but not in the looped heart tube. FHF, first heart field; SHF, second heart field. Scale bar: 100 μm.

The cardiac malformations exhibited in *Hnrnpa1^ct/ct^* homozygous mutant mice led us to investigate the role of *Hnrnpa1* during cardiac development. We performed whole-mount in situ hybridization on wild-type embryos from E7.0–E9.5 using riboprobe complementary for the 3′ UTR region of *Hnrnpa1* mRNA (Figure 3). We first used this riboprobe to hybridize E9.5 wild-type, *Hnrnpa1^+/ct^*, and *Hnrnpa1^ct/ct^* embryos from the same litter. No signal was detected in the *Hnrnpa1^ct/ct^* homozygous mutants, which demonstrated loss of *Hnrnpa1* transcripts and the specificity of this riboprobe (Supplemental Figure 6). *Isl1* was stained as an indicator for the SHF, while *Nkx2.5* was also stained to indicate both first heart field (FHF) and second heart field (SHF) cardiac progenitors. In the pre–cardiac crescent stage (Figure 3, A and B), expression of *Hnrnpa1* mRNA was detected in the pre–cardiac ALPM. In the ensuing cardiac crescent stage, *Hnrnpa1* mRNA was expressed in both the FHF and SHF, from which the myocardial cells and cardiac progenitors in the splanchnic mesoderm are derived, respectively, compared with the expression patterns of *Isl1* and *Nkx2.5* (Figure 3, C–E). After heart tube formation, while a strong expression of *Hnrnpa1* was maintained in the splanchnic mesoderm, which was partially overlapped with the *Isl1*-expressing domain, the expression of *Hnrnpa1* was almost lost in the *Nkx2.5*-expressing differentiated myocardium (Figure 3, F–H). Following with the initiation of the cardiac looping process, *Hnrnpa1* continued to be strongly expressed in the splanchnic mesoderm of the dorsal pericardial wall, spanning from the distal OFT to the sinus venosus, but was absent in the myocardium of the looped heart tube. The strongest expression was detected in the left and right lateral sides of the splanchnic mesoderm (Figure 3I). At E9.5, the *Hnrnpa1*-expressing domain in the splanchnic mesoderm overlapped with the region of the SHF.

### Hnrnpa1^ct/ct^ mutation leads to loss of Hnrnpa1 expression and dysregulation of both SHF- and heart tube–specific cardiac genes at E9.5.

**Figure 4:**
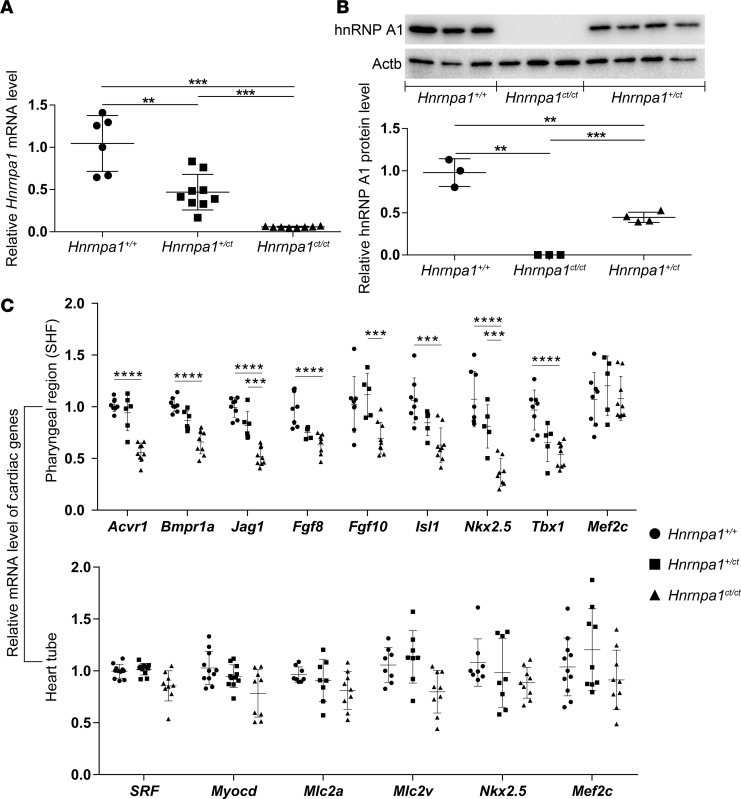
Expression of *Hnrnpa1* and relative cardiac genes in *Hnrnpa1^ct/ct^* mutant embryos at E9.5. *GAPDH* was used as the internal control for qRT-PCR, and β-Actin was used as the loading control for Western blot, respectively. (**A**) qRT-PCR results for E9.5 embryos. The *Hnrnpa1* mRNA level in all 3 genotypes is shown. Six wild-type littermate controls, nine heterozygous mutants, and eight homozygous mutants were used. (**B**) Three wild-type littermate controls, three homozygous mutants, and four heterozygous mutants were used for Western blot analysis. (**C**) Total RNA was extracted from isolated pharyngeal region or heart tube respectively at E9.5. qRT-PCR was performed to monitor the expression of both the second heart field (SHF) and heart tube–specific cardiac genes. The number of *Hnrnpa1^+/+^*, *Hnrnpa1^+/ct^*, and *Hnrnpa1^ct/ct^* embryos in the SHF: 8, 5, and 9 for *Fgf8*, *Fgf10*, *Isl1*, *Mef2c*, and *Tbx1*; 8, 5, and 8 for *Nkx2.5*; 8, 6, and 9 for *Acvr1*, *Bmpr1a*, and *Jag1*, respectively. The number of *Hnrnpa1^+/+^*, *Hnrnpa1^+/ct^*, and *Hnrnpa1^ct/ct^* embryos in the heart tube: 8, 8, and 9 for *Mlc2a*, *Mlc2v*, and *Nkx2.5*; 11, 9, and 9 for *Mef2c*; 11, 11, and 9 for *Myocd* and *SRF*, respectively. The values represent mean ± SD in independent samples. **P < 0.01, ***P < 0.001, ****P < 0.0001 by unpaired 2-tailed *t* tests with Bonferroni correction.

The 2-base deletion in *Hnrnpa1* in mice was predicted to cause a frameshift mutation and to stop of the translation early, leading to the nonsense-mediated decay of the transcript. We quantified the expression of *Hnrnpa1* mRNA in mutants of *Hnrnpa1* and discovered that E9.5 homozygous mutants (*Hnrnpa1^ct/ct^*) displayed only about 5.86% of the expression level compared with wild-type embryos, whereas the relative expression of heterozygous whole embryos (*Hnrnpa1^+/ct^*) comprised about 42.7% of the expression, as measured by quantitative RT-PCR (qRT-PCR) (Figure 4A). This result is consistent with the previous whole-mount in situ hybridization analysis (Supplemental Figure 6). Western blot analysis was also performed for all 3 genotypes at E9.5. The hnRNP A1 protein was not examined in *Hnrnpa1^ct/ct^* mutants, whereas only 45.7% of hnRNP A1 expression was detected in *Hnrnpa1^+/ct^* mutants compared with wide-type littermate controls (Figure 4B). The 2-base deletion leads to loss of *Hnrnpa1* in both mRNA and protein levels.

The pharyngeal tissues (SHF) or heart tubes of E9.5 embryos were isolated separately and used to examine the transcription level of SHF- or heart tube–specific cardiac genes by qRT-PCR, respectively (Supplemental Figure 7). For the SHF lineage, we analyzed the expression levels of two bone morphogenetic protein (BMP) type I receptors, *Acvr1* and *Bmpr1a*; two kinds of fibroblast growth factor (FGF) ligands, *Fgf8* and *Fgf10*; Notch ligand *Jagged1* (*Jag1*); and cardiac transcription factors, including *Isl1*, *Mef2c*, *Nkx2.5*, and *Tbx1*. All of these genes have been confirmed to regulate development of the SHF. In the heart tube, we also investigated the expression levels of *Mef2c*, *Mlc2a*, *Mlc2v, Myocd*, *Nkx2.5*, and *SRF*, as these genes played important roles during cardiomyocytes differentiation. The mRNA levels of several SHF cardiac markers, including *Acvr1*, *Bmpr1a*, *Fgf8*, *Isl1*, *Jag1*, *Nkx2.5*, and *Tbx1* decreased significantly in the pharyngeal region of *Hnrnpa1^ct/ct^* mutants. However, no significant difference in *Mlc2a*, *Mlc2v, Myocd*, and *SRF* transcripts was detected in the *Hnrnpa1^ct/ct^* mutant heart tube. The transcripts of *Nkx2.5* reduced significantly in the pharyngeal region of *Hnrnpa1^ct/ct^* mutants but not in the heart tube. No significant change in *Mef2c* expression was detected in both the SHF and the heart tube (Figure 4C). These results demonstrate that this *Hnrnpa1^ct/ct^* mutation leads to dysregulation of a series of SHF-specific cardiac genes, which should be responsible for cardiac looping defects exhibited in the *Hnrnpa1^ct/ct^* mutants at E9.5.

### Altered expression pattern of Nkx2.5 and Isl1 demonstrates cardiac defects associated with both FHF and SHF lineages in Hnrnpa1^ct/ct^ homozygous mutants.

The expression pattern of *Hnrnpa1* is reminiscent of that of other essential genes during cardiac development. The VSD, PTA, and DORV displayed in the *Hnrnpa1^ct/ct^* homozygous mutants suggest that *Hnrnpa1* may be involved in the proliferation/migration of cardiac progenitors. To investigate the effect of *Hnrnpa1* deletion on other cardiac developmental genes, whole-mount in situ hybridization was performed on E8–E9.5 embryos to analyze the expression pattern of *Nkx2.5* and *Isl1*.

**Figure 5:**
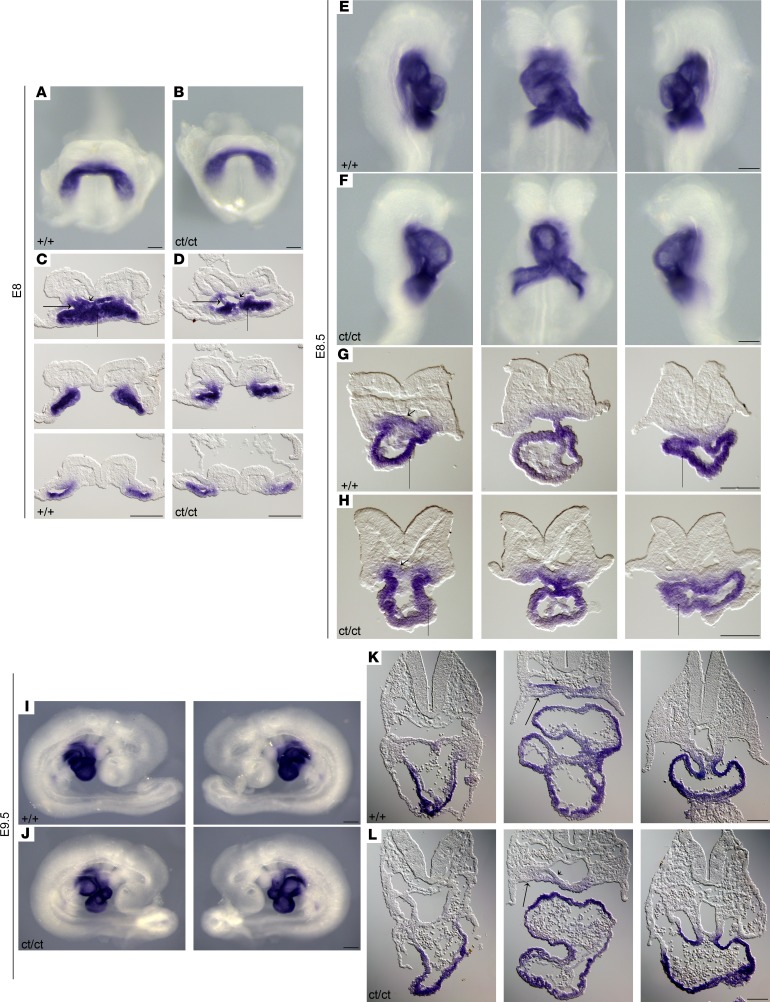
In situ analysis for *Nkx2.5* demonstrates that *Hnrnpa1^ct/ct^* mutation leads to severe defects in both the FHF and SHF lineages. Detection of *Nkx2.5* mRNA by whole-mount in situ hybridization in both wild-type littermate controls and *Hnrnpa1^ct/ct^* homozygous mutants. Wild-type littermates and homozygous mutants are indicated by +/+ and ct/ct, respectively. Sections shown below are from arterial pole to venous pole. At each stage, results from 1 of 3 representative experiments are displayed. (**A–D**) E8 (3 somite pairs) embryos are shown in the ventral view, with corresponding sections. (**E–H**) E8.5 (9 somite pairs) embryos are shown from the right, ventral, and left, with sections from anterior to posterior poles. (**I–L**) E9.5 (25 somite pairs) embryos are shown from right to left lateral views, with corresponding sections. The arrows in each photo represent the regions with lower *Nkx2.5* mRNA levels in homozygous mutants. A markedly changed expression pattern of *Nkx2.5* was detected in differentiated myocardium (thin long arrow), foregut endoderm (short arrow), and splanchnic mesoderm (SHF, wide long arrow). FHF, first heart field; SHF, second heart field. Scale bar: 100 μm.

*Nkx2.5* is reportedly expressed in both the FHF and SHF. In the heart tube, *Nkx2.5* can either cooperate with *Tbx5* or work with *Tbx2* and *Tbx3* to promote or suppress the differentiation of myocardium, respectively (25–27). Recent work also shows that *Nkx2.5* is essential in coordinating the proliferation and differentiation in the SHF (28, 29). Whole-mount in situ analysis demonstrated that CT deletion in *Hnrnpa1* alters the expression pattern of *Nkx2.5* mRNA at several critical developmental time points (Figure 5). At the cardiac crescent stage (3 somite pairs), there was a marked loss of *Nkx2.5*-expressing myocardium in the cardiac crescent (FHF lineage) of the *Hnrnpa1^ct/ct^* homozygous mutants (Figure 5, C and D, thin long arrow). Reduced expression of *Nkx2.5* mRNA was also detected in the medial splanchnic mesoderm (SHF lineage) (Figure 5, C and D, wide long arrow) as well as in the ventral foregut endoderm (Figure 5, C and D, short arrow). After the initiation of cardiac looping (9 somite pairs), a lower expression level of *Nkx2.5* mRNA was detected in both the OFT and sinus venosus myocardium (Figure 5, G and H, thin long arrow) as well as in the ventral foregut endoderm at the arterial pole (Figure 5, G and H, short arrow). *Hnrnpa1^ct/ct^* homozygous mutant hearts were truncated and resembled the primitive linear heart tube, with posterior extension of the *Nkx2.5*-expressing sinus horns (Figure 5, E and F). By late E9.5 (25 somite pairs), the *Hnrnpa1^ct/ct^* mutant heart was incompletely compacted, with enlarged atria (Figure 5, I and J). The expression of *Nkx2.5* in the splanchnic mesoderm was confined to the more medial region (Figure 5, K and L, wide long arrow), together with a reduced level in the ventral foregut endoderm (Figure 5, K and L, short arrow). These results suggest that *Hnrnpa1* may be required for the maintenance or proliferation of *Nkx2.5*-positive cardiac progenitors derived from both the FHF and SHF lineages.

**Figure 6:**
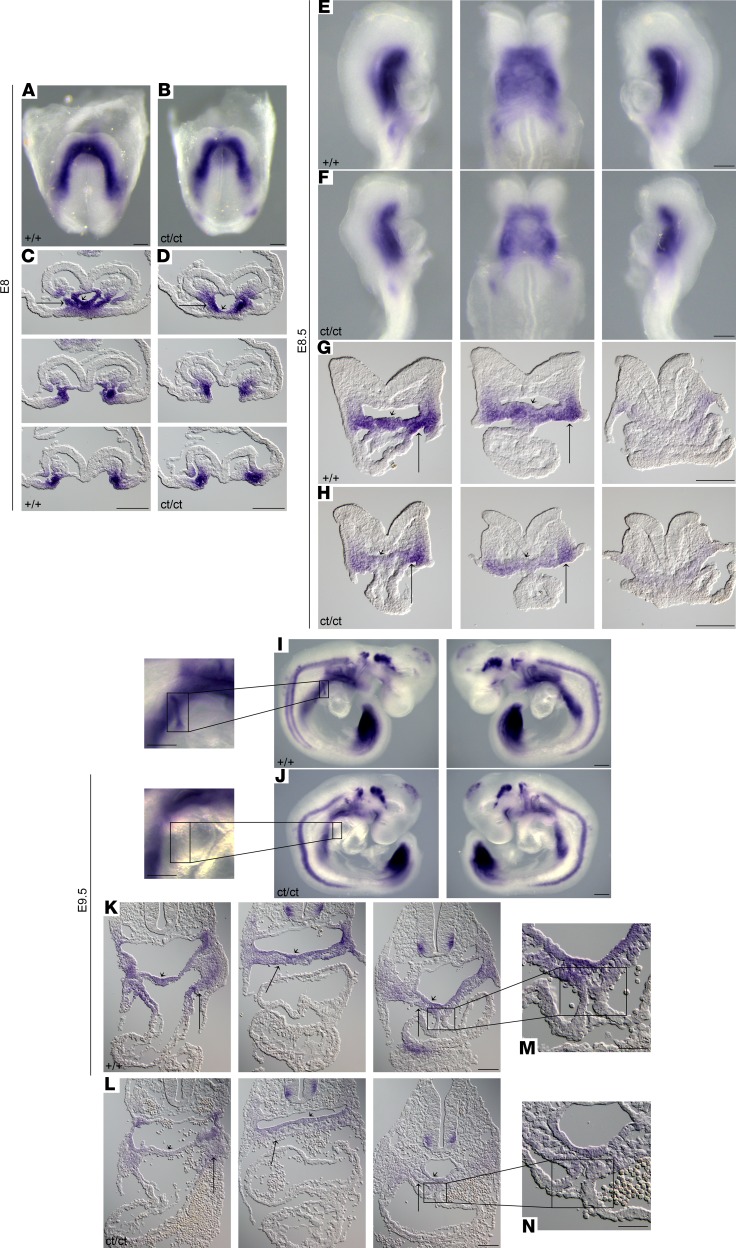
Altered expression pattern of *Isl1* shows SHF-related defects in *Hnrnpa1^ct/ct^* mutants. Whole-mount in situ hybridization analysis of *Isl1* mRNA in *Hnrnpa1^ct/ct^* homozygous mutants and wild-type littermate controls. Wild-type embryos and *Hnrnpa1* homozygous mutants are labeled by +/+ and ct/ct, respectively. At each stage, results from 1 of 3 representative experiments are displayed. (**A**, **B**, **E**, **F**, **I**, and **J**) Whole-mount in situ hybridization analysis for embryos at E8 (2 somite pairs), E8.5 (9 somite pairs), and E9.5 (25 somite pairs). (**I** and **J**) The expression of *Isl1* in a small subset of SAN progenitors is lost in *Hnrnpa1^ct/ct^* homozygous mutants. (**C**, **D**, **G**, **H**, **K**, and **L**) Corresponding sections are shown below at different levels from arterial pole to venous pole. (**C** and **D**) Sections at the cardiac crescent stage (E8). (**G** and **H**) Sections are shown at OFT region, middle portion of the heart tube, and sinus venosus (E8.5). (**K** and **L**) Sections are shown from OFT to sinus venous at E9.5. The expression of *Isl1* decreases markedly in the truncated DM of *Hnrnpa1^ct/ct^* homozygous mutants. (**M** and **N**) Decreased *Isl1* mRNA or *Isl1*-expressing cardiac progenitors can be found in both splanchnic mesoderm (SHF, wide long arrow) and ventral foregut endoderm (short arrow). DM, dorsal mesocardium; OFT, outflow tract; SAN, sinoatrial node; SHF, second heart field. Scale bar: 100 μm.

We next examined *Isl1*, which marks the SHF lineage (30, 31). *Isl1* can either cooperate with *Mesp1* to induce the specification of earliest cardiac progenitors or promote proliferation and maintain differentiation delay in the SHF. *Isl1* also coordinates several signaling pathways, including the canonical WNT, FGF, and BMP pathways (31–34). Whole-mount in situ analysis of *Isl1* mRNA showed severe SHF associated defects in the *Hnrnpa1^ct/ct^* homozygous mutants (Figure 6, A–L). At the cardiac crescent stage (2 somite pairs), the *Isl1*-expressing domain in the medial splanchnic mesoderm was restricted to a more anterior region along the body axis compared with the wild-type embryos (Figure 6, A and B). Formation of pharyngeal mesoderm (SHF lineage) (Figure 6, C and D, wide long arrow) and foregut endoderm (Figure 6, C and D, short arrow) were also impaired. Further results showed that *Isl1* expression in both splanchnic mesoderm (Figure 6, G and H, wide long arrow) and ventral foregut endoderm (Figure 6, G and H, short arrow) was progressively lost after the initiation of cardiac looping morphogenesis in the E8.5 (9 somite pairs) embryos and was more marked in the arterial pole. At E9.5 (25 somite pairs), *Isl1* expression was markedly reduced throughout the dorsal pericardial wall spanning from the OFT to the sinus venosus in both splanchnic mesoderm (Figure 6, K and L, wide long arrow) and foregut endoderm (Figure 6, K and L, short arrow). By E9.5, *Isl1* was expressed in a small population of cells in the right lateral junction between the atria and sinus horn to initiate the specification of sinoatrial node (SAN) progenitors (35). Such expression was absent in the *Hnrnpa1^ct/ct^* mutants, indicating the impaired development of the SAN (Figure 6, I and J). Moreover, the expression of *Isl1* decreased substantially in the dorsal mesocardium (DM) (Figure 6, M and N), which might interrupt the development of dorsal mesenchymal protrusion (DMP) and atrioventricular septum in later stages (36).

*Nkx2.5* and *Isl1* play an important role to coordinate the specification, maintenance, and proliferation of cardiac progenitors. The altered expression pattern of *Nkx2.5* and *Isl1* in cardiac progenitors suggests that the severe congenital cardiac defects associated with *Hnrnpa1^ct/ct^* mutation may be mediated by genetic regulation of *Nkx2.5* and *Isl1*.

### KO of Hnrnpa1 prevents the differentiation of mESCs into cardiomyocytes in vitro.

**Figure 7:**
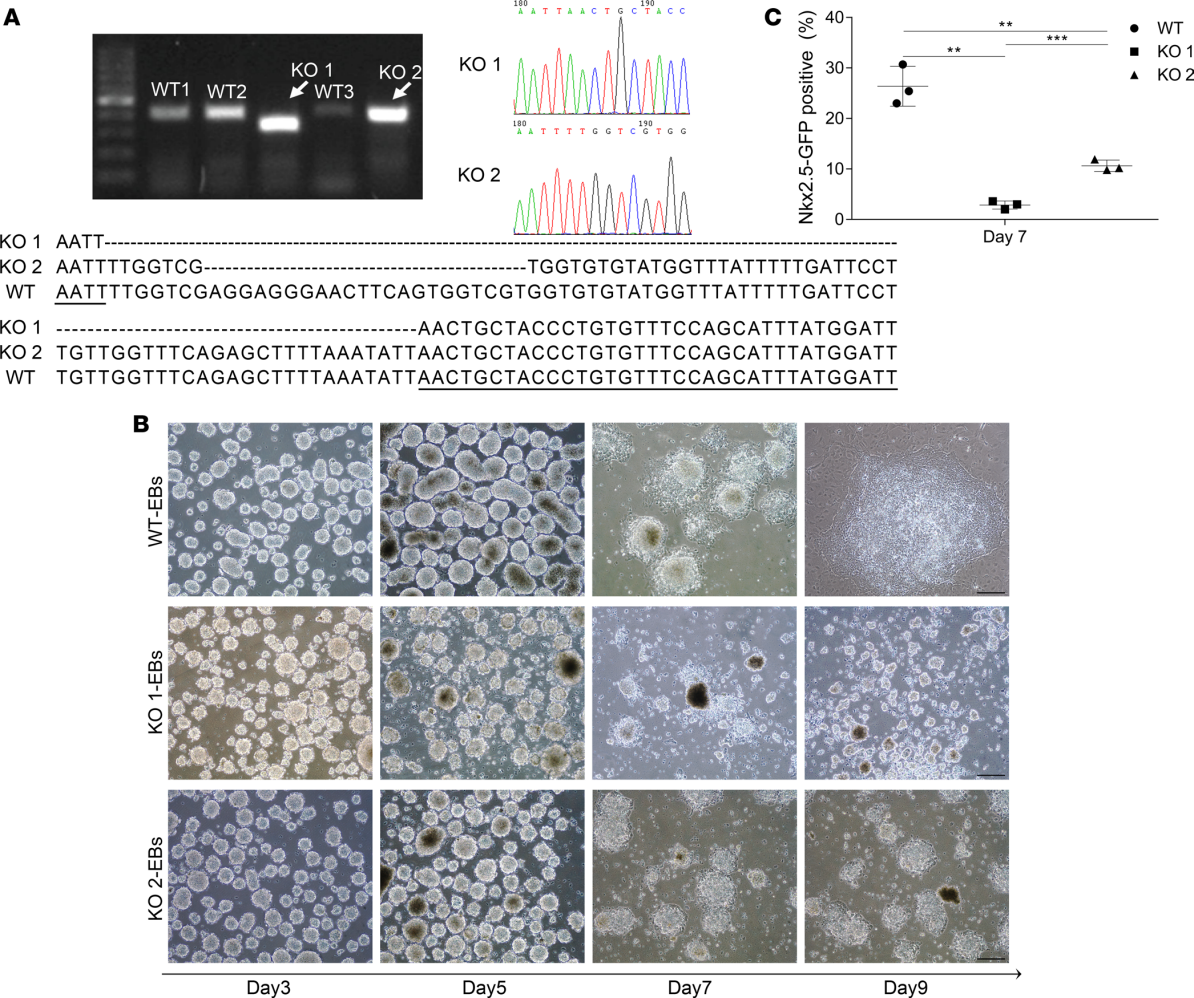
KO of *Hnrnpa1* impaired cardiomyocyte differentiation in vitro. (**A**) *Hnrnpa1* KO 1 and KO 2 mESC lines were established by the CRISPR/Cas9 method. PCR products of exon 6 in Nkx2.5-EGFP-mESCs (lane 2, 3 and 5, 425 bp), *Hnrnpa1* KO 1 (lane 4, 343 bp), and KO 2 (lane 6, 404 bp) were shown. Sequencing and blasting results demonstrate that 82-bp and 21-bp fragments of exon6 of *Hnrnpa1* are deleted in *Hnrnpa1* KO 1 and KO 2, respectively, by the CRISPR/Cas9 technique. (**B**) For each group, in vitro differentiation was performed 3 times. mESCs were induced into cardiomyocytes. Embryonic bodies (EBs) formed 2–3 days later. Loosely aggregated EBs were observed in *Hnrnpa1* KO 1 and KO 2 groups (day 3–5). After attachment of EBs to the plate at day 6, outgrowths were impaired in both *Hnrnpa1* KO 1 and KO 2 groups (day 7–day 9). Scale bar: 200 μm. (**C**) The percentage of Nkx2.5-EGFP–positive cells was analyzed by flow cytometry at day 7. Compared with the Nkx2.5-EGFP-mESC group, significantly reduced Nkx2.5-EGFP–positive cells were detected in both *Hnrnpa1* KO 1 and KO 2 groups. Data in **C** are presented as mean ± SD, with *n* = 3 per group. **P < 0.01; ***P < 0.001 by unpaired 2-tailed *t* tests with Bonferroni correction.

*Hnrnpa1* KO 1 and KO 2 mouse embryonic stem cells (mESCs) were generated by CRISPR/Cas9 deletion of different fragments of exon 6 of *Hnrnpa1* in Nkx2.5-EGFP-mESCs (Figure 7A). A single mESC was seeded into ultra-low attachment plates, and formation of embryonic bodies (EBs) was observed 2–3 days later. Between day 3 and day 5, EBs from Nkx2.5-EGFP-mESCs formed tight clusters, whereas EBs in *Hnrnpa1* KO 1 and KO 2 groups were loosely aggregated (Figure 7B). At the end of day 6, EBs in all groups were transferred into a new fibronectin-coated plate separately to initiate cardiomyocytes differentiation. Almost all EBs in the Nkx2.5-EGFP-mESC group attached to the plate and formed outgrowths between day 6 and day 9. In contrast, EBs of *Hnrnpa1* KO 1 and KO 2 mESCs attached and outgrew poorly at a slower rate, with obviously elevated cell death. Only very few living cells could be observed in KO 1 and KO 2 groups at day 9 (Figure 7B). mESCs in all groups were collected at day 7 to assess the status of cardiomyocytes differentiation. Flow cytometry analysis demonstrated that significantly fewer Nkx2.5-EGFP–positive cells were present in *Hnrnpa1* KO 1 and KO 2 culture compared with the Nkx2.5-EGFP-mESC group (Figure 7C and Supplemental Figure 8). All mESCs in KO 1 and KO 2 groups died after day 9.

### Heterozygous mutations in HNRNPA1 in human CHDs.

**Figure 8:**
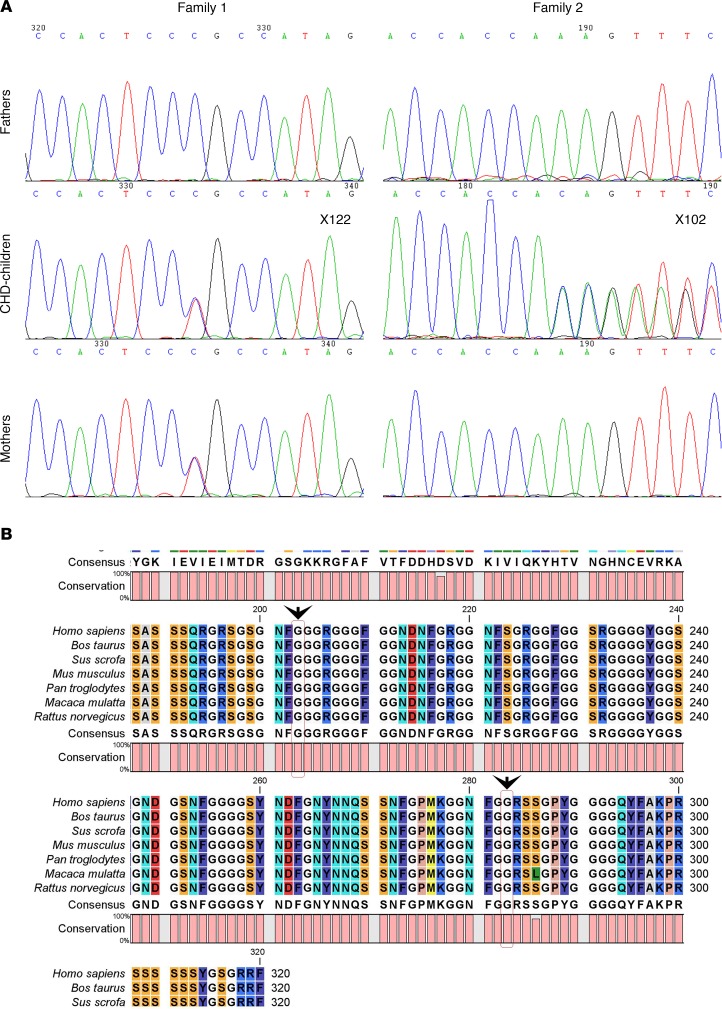
*HNRNPA1* mutations in human congenital heart disease patients. (**A**) Mutations in 2 trio families. (**B**) Both Gly^203^ and Gly^283^ locate in the conserved region of HNRNPA1 protein. CHD, congenital heart disease.

We sequenced human *HNRNPA1* in two sample sets containing 273 Chinese CHD trio probands and 225 Pakistani probands with CHD (Supplemental Tables 3 and 4). A rare missense variant (NM_031157.2, c. G847A; NP_112420.1, p. G283R, rs375259222) was identified in a 5-month-old boy (ID X122) with VSD, a history of patent foramen ovale, moderate mitral regurgitation, and pulmonary hypertension. A novel indel variant (NM_031157.2, c. 607-609 del GGT; NP_112420.1, p. 203 del G) in a 1-year-old boy (ID X102) with VSD (infracristal L–R) was also detected. We also sequenced the gene of available parents. The patient (ID X122) with the p.G283R variant (rs375259222) inherited this variant from his mother, and clinical information about his mother was not available. The patient (ID X102) with the p.203 del G harbored a de novo deletion (Figure 8A).

We considered how the variants potentially affect the protein. rs375259222 (G283R) located after the RRM superfamily domain with a nonpolar amino acid glycine was replaced by a polar positive amino acid arginine at position 283. The de novo mutation of p. 203 del Gly is located in the poly-Gly region that is important for protein structure and not near the C-terminus. Gly^203^ and Gly^283^ are both highly conserved among all species (human, cattle, pig, mice, chimpanzee, monkey, and rat; Figure 8B). Mutation Taster, PolyPhen 2, FATHMM, and other 4 programs predicted the variant (G283R) of rs375259222 as possibly damaging (Table 2).

These two variants were not found in NCBI dbSNP (version 144), in the 1000 genome project database, or in our 300 normal controls; rs375259222 is in the EVA_EXAC database (2015 version), with 7 allele counts among the 103,578 total allele counts, indicating that it is a very rare allele in populations. These 7 allele counts are all found in Asians, and it is most likely a recent rare variant.

## Discussion

*IIA*^−/−^ mice displayed complex congenital malformations, ranging from heart malformations and head truncation, resulting in prenatal lethality to near normal phenotype. The variability of the phenotype was shown to be determined by a genetic element that is approximately 9 cM away from the *Col2a1* locus. Our linkage analysis revealed that the CHD-causing locus is linked with the mutant *IIA* locus on chromosome 15 and originates from the 129S1/SvImJ allele in the R1 ES cell line. Upon crossing over with the allele from C57BL/6N, 129/SvEv, or ICR, the phenotype was lost. Our fine mapping using a congenic set of C57-*IIA*^−/−^ mice narrowed down the candidate region to 1.2 Mb (from chr15:102.29–103.49 Mb) and showed a recessive and simple Mendelian inheritance pattern of the new genetic locus. A frameshift mutation of the *Hnrnpa1* gene was identified to cause CHD phenotypes in mouse. *Hnrnpa1* mRNA was almost absent in the *Hnrnpa1^ct/ct^* homozygous mutant, caused by the nonsense-mediated decay of the transcripts’ machinery. Loss of *Hnrnpa1* expression leads to cardiac looping defects at E9.5, VSD and PTA at E13.5, and embryonic lethality at P0, or VSD/DORV in some rare cases. *Hnrnpa1* is expressed in both FHF and SHF cardiac lineages during the cardiac crescent stage; however, *Hnrnpa1* was only strongly maintained in SHF progenitors after heart tube formation in mouse embryos. *Hnrnpa1* mutant mice have reduced *Hnrnpa1* mRNA expression in a dose-dependent manner. The *Hnrnpa1^ct/ct^* mutation leads to altered expression pattern of *Nkx2.5* and *Isl1*.

### Hnrnpa1 is involved in the development of both the FHF and SHF.

The heart originates from two different lineages, the FHF and the SHF. The earliest differentiated cardiomyocytes are generated in the ALPM (37, 38). Then, the differentiated myocardial cells, which are situated in the lateral splanchnic mesoderm, form the cardiac crescent, whereas the SHF-derived cardiac progenitors are localized medially and dorsally to these differentiated myocardial cells. Whole-mount in situ analysis demonstrates that *Hnrnpa1* mRNA is expressed within ALPM in the pre–cardiac crescent stage. During the cardiac crescent stage, *Hnrnpa1* is strongly expressed in both the splanchnic mesoderm and differentiated myocardium. Reduction of *Nkx2.5*-expressing myocardium (Figure 5, C and D) and loss of *Isl1*-expressing tissues around the pharyngeal region in *Hnrnpa1^ct/ct^* homozygous mutants (Figure 6, C and D) support the role of *Hnrnpa1* in both FHF and SHF lineages during early cardiac development. Similar to loss of *Nkx2.5*-expressing myocardium, the reduced *Nkx2.5*-expressing population was also observed during the differentiation of *Hnrnpa1*-KO mESCs into cardiomyocytes (Figure 7C). After the formation of primitive heart tube, *Hnrnpa1* maintains strong expression in the splanchnic mesoderm but not in the heart tube. Though the primitive heart tube forms in *Hnrnpa1^ct/ct^* homozygous mutants, the elongation and looping process of the heart tube was not initiated till E8.5 (9 somite pairs) (Figure 5, E and F). A truncated and incomplete looped heart tube was detected at E9.5 (25 somite pairs; Figure 5, I and J). Markedly reduced *Isl1* expression (Figure 6, K and L) and restricted *Nkx2.5*-expressing domain (Figure 5, K and L) in the splanchnic mesoderm indicate SHF-derived cardiac defects. Thus, *Hnrnpa1* may be essential for both FHF and SHF lineages at the cardiac crescent stage but plays a major role in the SHF after heart tube formation.

### Hnrnpa1^ct/ct^ mutation leads to perturbations of cardiac transcription networks and multiple signaling pathways, including the FGF, BMP, and Notch pathways, in the SHF.

During cardiac looping morphogenesis, SHF progenitors are recruited toward the distal OFT and venous region of the elongating heart tube, which finally contribute to the development of the OFT, right ventricle, both atria, and a small portion of the left ventricle (31, 39). Proper development of the SHF is essential for both remodeling of the OFT and cardiac septum formation. Impaired development of the SHF has been associated with OFT alignment defects, such as DORV, PTA, TGA, and TOF (40–43). Other evidence shows that disturbance of regulatory networks in the SHF leads to a variety of VSDs at the arterial pole and causes a series of ASDs or AVSDs at the venous pole (44–49). Thus, the VSD, PTA, and DORV displayed in the *Hnrnpa1^ct/ct^* homozygous mutants seem to be associated with impaired SHF development.

The development of the SHF is regulated by complex transcription networks. qRT-PCR results demonstrate that the expression levels of several cardiac transcription factors, including *Isl1*, *Nkx2.5*, and *Tbx1*, decreased significantly in the SHF of *Hnrnpa1^ct/ct^* homozygous mutants (Figure 4C). All of these transcription factors have been implied in the pathogenesis of CHDs. Failure of cardiac looping morphogenesis has been observed in both *Isl1-* and *Nkx2.5*-null mouse models (31, 50). Specific inactivation of *Nkx2.5* in the SHF leads to complete penetrance of OFT defects, and PTA is the major phenotype (30). *Nkx2.5* mutations have been associated with a broad spectrum of human CHDs, including ASD, AVSD, DORV, TGA, TOF, VSD, and truncus arteriosus (7, 51–54). *Tbx1* is also proposed as a major candidate for del22q11.2 deletion syndrome (or DiGeorge syndrome), characterized by serious craniofacial and cardiovascular defects, including TOF and common arterial trunk (CAT) (6, 55). *Tbx1* heterozygous null mice also display a high incidence of OFT anomalies (56).

Interrupted signaling pathways in the SHF are also suggested to be involved in the pathogenesis of CHDs. qRT-PCR results also showed that *Hnrnpa1^ct/ct^* homozygous mutation impairs multiple signaling pathways, such as FGF, BMP, and Notch pathways (Figure 4C). FGF signaling has been proved to regulate the proliferation of SHF cardiac progenitors. Inactivation of *Fgf8* in the AHF leads to reduced proliferation and excess cell death in the pharyngeal mesoderm and adjacent tissues (57, 58). Impaired septation, rotation, and alignment of the OFT, including PTA, TGA, and DORV, as well as interrupted cardiac septal and valve development, such as VSD, have been observed in *Fgf8*-hypomorphic mice (59). Ablation of mesodermal *Fgf8* with *MesP1Cre* leads to hypomorphic OFT and right ventricle, which can be significantly exacerbated by compound inactivation of mesodermal *Fgf10* (60). BMP signaling has been proposed to inhibit FGF signaling, and the balance between FGF and BMP signaling is essential for regulation of proliferation and differentiation in the SHF cardiac progenitors (61, 62). In mouse embryos, conditional deletion of *Bmpr1a* via *Isl1-Cre* leads to CHDs, including ASD, VSD, PTA, and underdeveloped valves (63). Specific inactivation of *Bmpr1a* from venous pole SHF also leads to hypoplasia of the DMP precursors, failure of the DMP formation, and ostium primum defects, which contribute to the pathogenesis of AVSD (64). Deletion of *Acvr1* in the AHF can cause VSD and aorticopulmonary (AP) trunk septation defects (65). Notch signaling also plays a vital role during cardiac development, and absence of Notch ligand Jagged1 (Jag1) in the SHF causes severe cardiac anomalies, such as DORV and VSD (66).

Similar to these cardiac defects mentioned above, failure of looping morphogenesis, impaired cardiac septum formation (VSD), and OFT anomalies (PTA and DORV) have been observed in *Hnrnpa1^ct/ct^* homozygous mutants (Figure 2 and Supplemental Figure 5). qRT-PCR results demonstrated that expression levels of *Acvr1*, *Bmpr1a, Fgf8*, *Isl1*, *Jag1*, *Nkx2.5*, and *Tbx1* decrease significantly in the SHF of *Hnrnpa1^ct/ct^* homozygous mutants (Figure 4C). Thus, *Hnrnpa1* might play an upstream regulatory role during cardiac development. *Hnrnpa1^ct/ct^* homozygous mutation leads to dysregulation of multiple cardiac transcription factors and signaling pathways in the SHF, which further contributes to the pathogenesis of CHDs, including OFT malalignment and cardiac septal defects displayed in *Hnrnpa1^ct/ct^* mutant mice.

After the formation of the primitive heart tube, *Hnrnpa1* maintains its expression in the SHF but not in the heart tube. In *Hnrnpa1^ct/ct^* homozygous mutants, though the expression level of *Nkx2.5* decreased dramatically in the SHF region, no significant difference was detected in the heart tube by qRT-PCR. Consistent with this, no significant variations of *Mlc2a*, *Mlc2v*, *Myocd*, and *SRF* expression in the heart tubes or *Mef2c* expression in both heart tubes and the SHF tissues could be observed (Figure 4C). During smooth muscle differentiation, hnRNP A1 has been suggested as a transcription factor to activate the transcription of *MEF2c*, *SRF*, and *Myocd* (14). Thus, *Hnrnpa1* may have different regulatory role during cardiomyocytes differentiation.

All above results demonstrate that at E9.5 *Hnrnpa1* has a major regulatory role in the SHF. hnRNP A1 is usually considered as a repressor for alternative splicing, which plays an important role in modulation of exon skipping. hnRNP A1 can also work as a transcription factor to activate the transcription of downstream targets. In addition, hnRNP A1 has been reported to regulate the nuclear export, stability, and alternative translation of the mRNA (9–13). Thus, hnRNP A1 may regulate downstream cardiac genes via these different manners. However, the detailed mechanisms of how hnRNP A1 regulates these genes are still largely unknown.

### Hnrnpa1^ct/ct^ homozygous mutation may impair the development of the DM and SAN progenitors.

Whole-mount in situ hybridization and section analyses show impaired development of the DM and markedly reduced *Isl1* mRNA in this region (Figure 6, M and N). The DMP, which represents the protrusion of discrete mesenchymal cells from the DM toward the atrial cavity, is derived from the *Isl1*-expressing SHF lineage and is associated with atrioventricular septal defects (46, 47, 67, 68). DMP resides as an integral component of the atrioventricular mesenchymal complex and undergoes mesenchymal-to-myocardial transition in an *Nkx2.5*-dependent manner (46). The DM defects and altered *Isl1* and *Nkx2.5* expression pattern may disturb the formation of DMP and, thus, impair atrioventricular septal development in *Hnrnpa1^ct/ct^* homozygous mutants.

A previous study has indicated the important roles of *Isl1* and *Nkx2.5* in SAN development. *Isl1* is expressed in the majority of cardiac pacemaker cells throughout the developmental of SAN. Lineage tracing and specific ablation of *Isl1* in SAN lineage indicate that *Isl1* is essential for the maintenance, proliferation, and function of SAN cells (69). Transcriptome and ChIP analysis demonstrated that *Isl1* is involved in the expression regulation of several genes associated with SAN development, such as *Ank2* and *Tbx3* (69). Around E9.5, the earliest SAN progenitors, a *Isl1-* and *Tbx18*-positive but *Nkx2.5*-negative population, were detected in the right lateral junction between the atria and sinus horn (36, 70). *Isl1* expression in this region is absent in the *Hnrnpa1^ct/ct^* mutants (Figure 6, I and J), implicating the loss or deficient development of SAN progenitors. *Nkx2.5* suppresses the formation of SAN to establish the boundary between atrial myocardium and SAN progenitors, which can be antagonized by Shox2 (71–73). A recent model shows that *Nkx2.5* directly binds to an enhancer of *Isl1* and inhibits its transcriptional activity, which is required for the determination of myocytes subtype identity (74). *Hnrnpa1^ct/ct^* homozygous mutation leads to posterior extension of the *Nkx2.5*-expressing domain in the sinus horns at E8.5 (Figure 5, E and F) and expansion of the *Nkx2.5*-expressing atria at E9.5 (Figure 5, I and J), thereby suggesting that the loss of *Isl1* expression in SAN progenitors may be associated with ectopic expression of *Nkx2.5* in the caudal venous pole. Thus, *Hnrnpa1* may play an essential role upstream of *Nkx2.5* and *Isl1* to regulate the formation of SAN.

### Hnrnpa1 mutations contribute to CHDs.

In our mouse model, CHDs, including incomplete looped heart tube at E9.5, VSD and PTA at E13.5, and VSD and DORV at P0, were only detected in the *Hnrnpa1^ct/ct^* homozygous mutants, while no obvious cardiac defect was observed in the heterozygous mutants. The number of heterozygous mutant newborns also approximated the expected value. According to a recent study, muscle development was analyzed in the *Hnrnpa1*-null mouse line (24). Though the CHD phenotypes were not analyzed, significantly higher heart rates and systolic pressures were detected in E18.5 *Hnrnpa1* heterozygous null mice. Mouse exon array tests demonstrated that the expression pattern of multiple muscle-related genes was affected in the heterozygous mutant E18.5 hearts. The alternative splicing pattern of several muscle development–related genes, including *Mef2c*, *Lrrfip1*, *Usp28*, and *Abcc9* was also changed, with a significantly lower full length/truncated transcripts ratio (24). These results indicated that both heterozygous and homozygous mutations of *Hnrnpa1* might have a role in the pathogenesis of CHDs.

Two heterozygous rare mutations of *HNRNPA1* in human CHDs were also detected. We sequenced the human *HNRNPA1* gene in 273 Chinese CHD trio probands and 225 sporadic Pakistani nonsyndromic CHD patients and discovered one rare inherited missense mutation and one de novo indel heterozygous nonsynonymous variant; both of these two cases mainly present the VSD phenotype with some additional complex heart phenotypes (Figure 8 and Supplemental Table 3). All this evidence supports the important role of hnRNP A1 in cardiac development in both mice and human. Mutations associated with CHDs have been identified in at least 15 transcription factor genes (e.g., *NKX2-5*, *GATA4*, *ILS1*, and *HAND2*); 17 receptors, ligands, and signaling genes (e.g., *ACVR2B*, *NOTCH1*, and *SMAD6*); and 5 structural protein genes (e.g., *ACTC* and *ELN*) (1, 5). More recently, mutations in cilia and cilia-transduced cell signaling pathway genes have been identified by using unbiased screening ethylnitrourea-mutagenized mice (75). Most of these known genes for CHDs are involved in the altered levels of developmental signaling molecules during cardiogenesis. However, an important role of the RNA-binding protein in embryonic heart development is suggested.

The human *HNRNPA1* encodes a full-length 372–amino acid protein (accession NP_112420.1) and a truncated 320–amino acid isoform (accession NP_002127.1). Similar to the *Hnrnpa1^ct/ct^* mutant mice, VSDs were also observed in human CHD patients with *HNRNPA1* mutations. However, unlike the *Hnrnpa1^ct/ct^* mutant mice, humans with rare *HNRNPA1* mutations did not display OFT defect phenotypes: PTA or DORV. The phenotypic heterogeneity may be underpinned by the facts that the *Hnrnpa1^ct/ct^* mutation is located in the RRM domain, even though both mutations found in the human samples are located outside this domain (at the 203–amino acid and 283–amino acid positions, accession NP_112420.1), and that both of the human sample sets were found in live-born CHD patients with heterozygous mutations, suggesting that homozygous mutations might have caused more severe nonviable phenotypes, which were therefore not presented. Because the majority of *Hnrnpa1*^ct/ct^ mice died before birth, the 2 P0 mice in our samples were a rarity. It was noted that the missense rare variant was inherited from the mother of one of the patients, who had a nonreported cardiac phenotype. To our knowledge, CHDs sometimes reveal incomplete penetrance, and some mild CHDs can be self-corrected, thus becoming undetected in adulthood.

### Conclusion.

In conclusion, mutations in the *Hnrnpa1* gene cause CHDs in both humans and mice. *Hnrnpa1* is critical for both the FHF and SHF during early cardiac development. However, in later stages, *Hnrnpa1* only has a major role in the SHF. Loss of *Hnrnpa1* in the SHF leads to dysregulation of cardiac transcription factors, including *Isl1*, *Nkx2.5*, and *Tbx1*, as well as signaling pathways, such as the BMP, FGF, and Notch pathways. Thus, CHDs can be caused by impaired development of both the FHF and SHF lineages. This study provides insights into the mechanism by which *Hnrnpa1* becomes involved in the cardiac development.

## Methods

### Animals.

Three congenic mouse lines were established by repeatedly backcrossing the *IIA^+/−^* mutant mice to C57BL/6N, 129/SvEv, and ICR wild-type mice to obtain *IIA^+/−^* mutants with homogenous backgrounds. The *IIA^−/−^* mutants generated from the 3 mouse lines were named C57-*IIA^−/−^*, 129-*IIA^−/−^*, and ICR-*IIA^−/−^*, respectively. Construction of mouse lines, collection of embryos, and genotyping were performed as described in the supplemental materials (Supplemental Methods and Supplemental Figures 1–5 and 9).

### Identification of the CHD-causing genetic locus.

After collection of pedigrees, the CHD-causing genetic locus was searched by genome-wide linkage mapping, fine mapping, and high-throughput targeted sequencing and finally confirmed by Sanger sequencing, as described in the Supplemental Methods. All primers used for genotyping are listed in Supplemental Table 5.

### Characterization of CHDs in Hnrnpa1 mutant mice by histological and OPT analyses.

The E13.5 embryos were sectioned and then stained with hematoxylin and eosin. OPT was performed for P0 mice (76). See the Supplemental Methods for further details.

### Whole-mount in situ hybridization.

Digoxigenin-labeled riboprobes, anti-digoxigenin-AP antibody (11093274910; MilliporeSigma), and BM Purple reagent (11442074001; Roche) were used for whole-mount in situ hybridization, as described in the Supplemental Methods.

### qRT-PCR.

cDNA generated from total RNA of wild-type embryos and heterozygous and homozygous mutants was subjected to SYBR Green-based real-time PCR analysis, as described in the Supplemental Methods. All qRT-PCR primers are listed in Supplemental Table 6.

### Western blot analysis.

Mouse embryos were dissected at E9.5 to analyze the expression level of hnRNP A1. β-Actin was used as the loading control. Primary antibodies used were anti-mouse hnRNP A1 (sc-32301; Santa Cruz) and anti–β-actin (A2228; MilliporeSigma). The blots were detected using chemiluminescence, as described in the Supplemental Methods.

### Cardiac stem cell differentiation into cardiomyocytes in vitro.

CRISPR/Cas9 was used to KO *Hnrnpa1* in Nkx2.5-EGFP-mESCs (E14.RP11-88L12.Nkx2-5-EmGFP from MMRRC). Target and off-target primers are listed in Supplemental Table 7. These cells were differentiated into cardiomyocytes in vitro, and the Nkx2.5-EGFP–positive percentage was analyzed by flow cytometry, as described in the Supplemental Methods.

### Screen of HNRNPA1 variants in human patients.

273 Chinese nonsyndromic CHD patients and 225 sporadic Pakistani CHD patients were recruited. We also sequenced 300 Chinese normal individuals to ensure that the mutation was absent in the normal subjects (Supplemental Table 3 and 4). The phenotype of CHDs and genotyping analysis were performed as described in the Supplemental Methods.

### Statistics.

NPL was calculated using Simwalk2. The NPL_ALL and BLOCK statistics (for recessive mode) were selected to evaluate the significance of the reported linkage. TDT analysis using 40 markers was performed at E9.5 and P0. *P* < 0.001 (Figure 1, A and B) was considered significant. Significant differences in penetrance of the CHD phenotypes among *Hnrnpa1^ct/ct^, Hnrnpa1^+/ct^,* and *Hnrnpa1^+/+^* mice at selected time points were determined using Fisher’s exact test (*P* < 0.001 was considered significant). For qRT-PCR and Western blot analyses, F test was performed first. Statistical differences were then determined using the unpaired Student’s *t* test (2-tailed *P* value, either equal or unequal variances depending on F test). Figures were generated using GraphPad Prism 6 software. *P* < 0.0167 (Figure 4, A and B, and Figure 7C) or *P* < 0.00111 (Figure 4C) was considered significant after Bonferroni corrections. Otherwise, *P* values of less than 0.05 were considered significant. Graphs represent mean ± SD.

### Study approval.

The mouse study was approved by the Committee on the Use of Live Animal in Teaching and Research, University of Hong Kong (no. 1264-06, 3598-15). All mice were housed in environmentally controlled rooms in the Laboratory Animal Unit. Written informed consent was obtained from patients’ parents or guardians prior to inclusion in the study. The study protocols conformed to the ethical guidelines of the 1975 Declaration of Helsinki and were approved by the local ethics committee of the National Research Institute for Family Planning, Beijing, China (IRB no. 20101015), the University of Karachi (ref. no. KIBGE/ICE/35046/Sc/20/03/2007), and the University of Hong Kong (IRB no. UW 13-550).

## Author contributions

YQS, KSEC, PCS, PPLT, PLFT, and QL designed the research; ZY, PLFT, BW, JW, JTS, ZZ, FG, AWLL, SB, ALCH, YG, ND, ZGZ, YZ, DSMW, and SYYW performed the research; PLFT, BW, XZ, ZY, SB, YF, AWLL, AA, PYK, PCS, PPLT, KSEC, and YQS analyzed results; and ZY, PLFT, PPLT, BW, QL, KSEC, and YQS wrote the paper.

## Supplementary Material

Supplemental data

## Figures and Tables

**Table 2:**
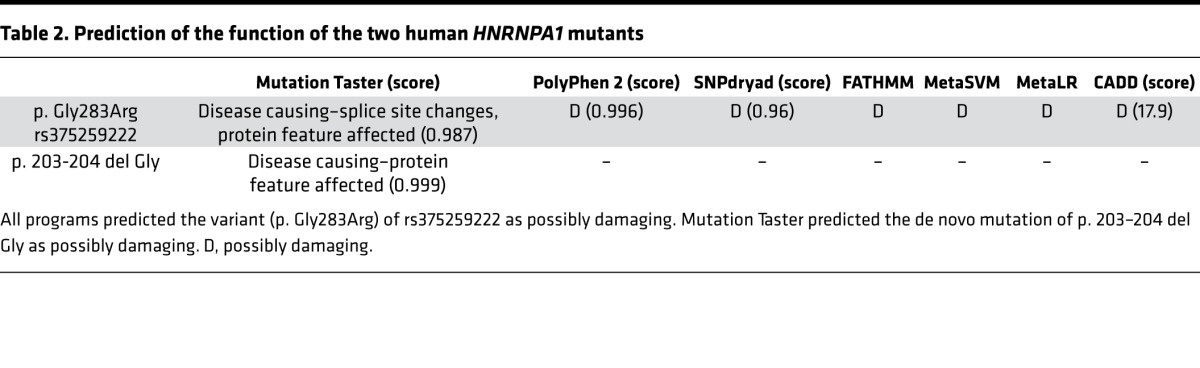
Prediction of the function of the two human *HNRNPA1* mutants

**Table 1:**
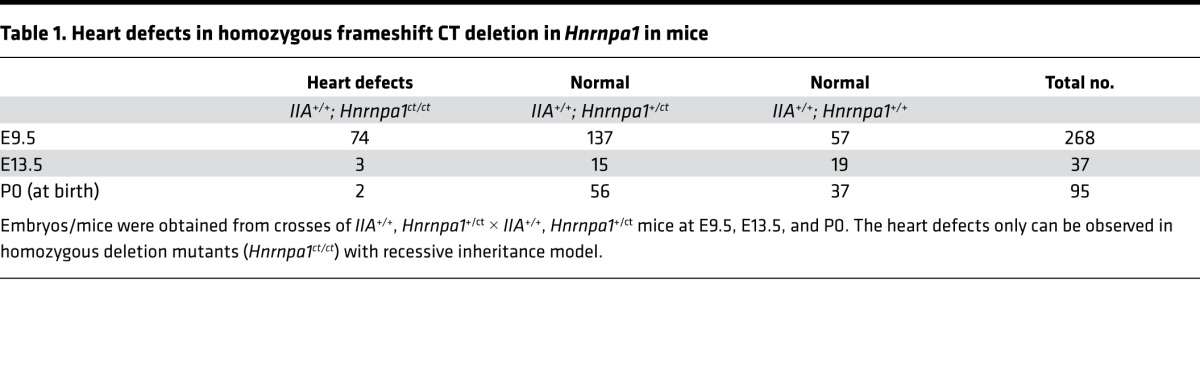
Heart defects in homozygous frameshift CT deletion in *Hnrnpa1* in mice

## Version 1 01/25/2018

Electronic publication
