# Supplementary material for: Mutations in Hnrnpa1 cause congenital heart defects
Source: JCI Insight. 2018 Jan 25;3(2):e98555. doi: 10.1172/jci.insight.98555 (PMC5821217; doi:10.1172/jci.insight.98555)
Supplement: Supplemental data [file jciinsight-3-98555-s001.pdf]

## Supplemental Methods

### Mouse embryo collection, phenotyping and DNA preparation

Embryos were harvested at embryonic day 7, 7.5, 8, 8.25, 8.5, 9.5, 13.5 and P0. At E9.5, E13.5 and P0, images of the left and right lateral sides of all the embryos were captured for CHD phenotype analyses. The morphologies of the hearts in both mutants and wild-type littermate controls were observed and compared.

Genomic DNA was extracted from the yolk sac or tissue by incubating in 40 µL digestion buffer (10 mM Tris-HCl with pH 8.0, 50 mM KCl, 0.5 % Tween-20, 0.5 % NP-40, and 60 mg of proteinase K) overnight at 55 °C followed by boiling at 95 °C for 10 min to inactivate proteinase K. For samples to be genotyped with SEQUENOM, DNA samples were diluted to 10-20 ng/µL and randomly assigned to a 96-well plate for genotyping.

### Genotyping of the *IIA* allele

To detect the genotype of the procollagen *IIA*-deficient mutant mice, two sets of primers (Set1: 5'Exon2 and OYY-KO3; Set2: mcol2intron1 and OYY-KO3; **Supplemental Table 5**) were used in a 25 µL reaction volume containing 10 x PCR buffer, 25 mM $MgCl_2$, 10 mM dNTPs, 100-200 ng DNA, and 1 U Taq. PCR reactions were carried out separately for the wild-type and mutant alleles. The reaction was hot-started for 4 min and run through 40 cycles with the following settings: 94 °C for 45 s, 55 °C for 45 s, and 72 °C for 45 s. PCR products from the wild-type and mutant alleles amplified using the different primer sets gave different combinations of bands upon gel electrophoresis. The wild-type products showed a band at 497 bp for Primer Set 1 and 665 bp for Primer Set 2; $IIA^{+/-}$ gave a band at 497 bp for Primer Set 1, and double bands at 665 and 474 bp for Primer Set 2; $IIA^{-/-}$ showed no band for Primer Set 1 and a single band at 474 bp for Primer Set 2.

### Collection of pedigrees for linkage mapping

Linkage mapping panel was constructed by intercrossing $IIA^{-/-}$ mutants (F1) collected from C57-$IIA^{+/-}$ x ICR $IIA^{+/-}$ crosses to generate a $IIA^{-/-}$ F2 population with segregation of the hypothetical heart defect allele (**Supplemental Figure 9**). The F2 population was collected at E9.5 and P0. E9.5 embryos were dissected from decidua in 1 x PBS. After dissection, extra-embryonic membranes were removed. The embryos were fixed in 4% PFA in 1 x PBS at 4 °C. Embryos were characterized and photographed. Abnormalities in heart looping were classified as the cases in subsequent linkage analysis. As for the newborns collected at P0, death was considered as a case. In total, 72 newborns (collected at P0), with 11 cases from 8 litters and 38 embryos (collected at E9.5) with 6 cases from 3 litters, were selected. Nonparametric linkage (NPL) was calculated using Simwalk2 (1). The NPL_ALL and BLOCK statistics (for recessive mode) were selected to evaluate the significance of the reported linkage.

### SNPs genotyping by SEQUENOM

SNPs were genotyped using the high-throughput SEQUENOM genotyping platform. Briefly, amplification and allele-specific extension primers were designed with MassARRAY® AsssayDesign software provided by SEQUENOM, San Diego, CA. PCR reactions were set up in 384-well plates at 6 µL total volume containing 5 ng genomic DNA, 0.3 pmol amplification primers, 200 mM dNTPs, 3.25 mM $MgCl_2$, and 0.2 U Hot-Star Taq polymerase (Qiagen, Valencia, CA). The reaction was hot started for 15 min and run through 45 cycles using the following settings: 95 °C for 20 s, 56 °C for 30 s, and 72 °C for 60 s, followed by 72 °C for 3 min. After PCR amplification, non-incorporated dNTPs were removed with shrimp alkaline phosphatase. The allele-specific extension primer immediately upstream from the polymorphic site was added together with a specific combination of deoxy dTTP and di-deoxy dATP, dCTP, dGTP, and thermosequenase (Amersham, Bioscience, Piscataway, USA). The extension products of a maximum of 40 polymorphic loci were then analyzed by using a modified Brucker Autoex MALDI-TOF mass spectrometer (Brucker, Billerica, MA). Genotyping was repeated in 10% of the samples for verification and quality

control. Poor DNA samples (with < 50% of the markers having genotyped) or poor sequencing markers (with < 50% of samples having genotyped) were filtered from the downstream analysis.

**Genotyping of mouse rs32183020 by Sanger sequencing**
Two primers (rs32183020-F and rs32183020-R; **Supplemental Table 5**) were first designed and amplified in a 50 μL reaction volume containing 10 x PCR buffer, 50 mM $MgCl_2$, 10 mM dNTPs, 100-200 ng DNA, and 1 U Taq. The reaction was hot-started for 4 min and run through 35 cycles using the following settings: 94 ℃ for 30 s, 55 ℃ for 30 s, and 72 ℃ for 30 s. The forward primer (rs32183020-F) was used for sequencing by using the ABI 3730XL sequencer.

**Targeted next-generation sequencing**
3 μg DNA was randomly fragmented and the both ends of fragments were ligated to adapters. Extracted DNA was then amplified by ligation-mediated PCR (LM-PCR), purified, and hybridized to the NimbleGen 2 and then washed out. Both the non-captured and captured LM-PCR products were subjected to q-PCR to estimate the magnitude of enrichment. Each captured library was then loaded on Illumina Hiseq2000 sequencer, and we performed high-throughput sequencing for each captured library to ensure that each sample met the desired average sequencing depth.

All the short reads were aligned against *Mus musculus* reference genome NCBI37/mm9 chr15 using BWA (2) version 0.5.9. Illumina 1.5 encoding Phred quality scores were converted to Sanger scores by BWA using the '-I' option. The multi-threading mode was enabled with eight threads concurrently generating SA coordinates for each mate-read and with permission of up to two mismatches. Picard tools (http://picard.sourceforge.net) version 1.66 was applied to prepare the original aligned results from BWA for variants detection in the succeeding step. First, mate-pair information was fixed to ensure that it is synced between each read and its mate pair with FixMateInformation function. Fixed BAM file generated from the original SAM file was then reordered and sorted using ReorderSam and SortSam tools, respectively. ReorderSam guaranteed that contigs in reads file are in ordered as those in the provided reference files, whereas SortSam sorted the order of the BAM file by coordinates (or unsorted, queryname are used as alternates). Finally, duplicates in the alignments were marked but not removed by Picard with MarkDuplicates model.

SAMtools (Sequence Alignment/Map) (3) version 0.1.17 with default parameters was used to detect SNPs and indels from the previous prepared BAM file. To avoid the possible overestimation of mapping qualities during BWA-short alignment, '-C50' option was enabled when we applied mpileup function in SAMtools. Results in bcf format from mpileup analysis were then translated into vcf file using bcftools. The maximum read depth (-D option) was set enlarged enough (2000) so as not to exclude any reads, as we would like to maximally cut down the false negatives.

ANNOVAR (4) version 20111120 was adopted for the functional annotation of the variants detected using SAMtools. The refSeq Gene and UCSC known genes were used for the gene-based annotation, whereas phastCons30way, tfbs, band, and segdup databases were downloaded from UCSC to carry out region-based annotation. We also mapped the variants to mouse dbSNP128 to identify the variants which were not recorded.

**Functional annotation of variants**

ANNOVAR (http://annovar.openbioinformatics.org/en/latest/),
SIFT (http://sift.jcvi.org/www/SIFT_BLink_submit.html),
PolyPhen (http://genetics.bwh.harvard.edu/pph2/),
MutationTaster (http://www.mutationtaster.org/)
SNPdryad (http://snps.ccbr.utoronto.ca:8080/SNPdryad/),

FATHMM (http://fathmm.biocompute.org.uk/) and CADD (http://cadd.gs.washington.edu/score) were used to annotate and predict the functional variants.

**Human subjects and *HNRNPA1* variants screening**

A total of 273 Chinese non-syndromic CHD patients and their parents with no reported cardiac phenotype and 225 sporadic Pakistani CHD patients were recruited for this study. Three hundred normal Chinese subjects were also recruited. All patients underwent an extensive clinical assessment, including standard physical examination, electrocardiogram (ECG), ultrasonic echocardiogram, and chest X-ray. We then classified them under different pathologic types according to the diagnosis mentioned above or open heart surgery. Familial histories and clinical information of all patients were reviewed.

The phenotypes of human CHDs included ventricular septal defect (VSD), pulmonary atresia (PA) or stenosis (PS), tetralogy of Fallot (TOF), atrial septal defect (ASD), patent ductus arteriosus (PDA), pulmonary hypertension (PH), and other complex cardiac malformations (**Supplemental Table 3**).

Human genomic DNA was extracted from peripheral blood leukocytes using standard methods. The human *HNRNPA1* gene is located on 12q13.13 and is encoded by eleven exons (NC_000012.12). The exons and nearby introns of *HNRNPA1* were amplified by PCR using specific primers (as shown in **Supplemental Table 4**). All fragments were sequenced using the ABI 3730XL sequencer. Meanwhile, PCR products were sequenced using the Sanger method.

When any mutation was detected in human CHD patients, we first searched the NCBI-SNP database to see whether the mutation existed or not, then we compared the observation with 1000 genome database. Finally, we sequenced 300 normal individuals to ensure that the mutation was absent in the normal subjects.

**Characterization of *Hnrnpa1* mutant mice by histological and OPT analyses**

The E13.5 embryos were sectioned and then stained with Hematoxylin and Eosin. Optical projection tomography (OPT) was performed for P0 mice (5). Hearts were dissected, placed in low-melt agarose, and exposed to benzyl alcohol/benzyl benzoate for imaging using a Bioptonics 3001 OPT Scanner (Edinburgh, UK). Images were visualized and evaluated using Volocity (Perkin Elmer, Waltham, MA).

***In situ* hybridization**

**Genotyping**

Wild-type and mutant embryos were harvested (E7, E7.5, E8, E8.25, E8.5, E9.5), and genomic DNA (NC_000081.6) was extracted from the yolk sac. PCR reactions were performed with the following primers *Hnrnpa1*-exon5-F and *Hnrnpa1*-exon5-R (**Supplemental Table 5**). The reaction was hot-started at 94 ℃ for 5 min and run through 35 cycles with the following settings: 94 ℃ for 30 s, 55 ℃ for 30 s, and 72 ℃ for 45 s. The wild-type or mutant alleles were then determined by sequencing.

**Whole mount *in situ* hybridization (WISH)**

WISH was performed with modification using a classical protocol previously described (6). References for digoxigenin (DIG)-labeled riboprobes used in this research are listed as below: *Nkx2.5* (7) and *Isl1* (8). The 551 bp *Hnrnpa1* riboprobe was generated by RT-PCR from mouse cDNA with primers 5'-CAGGAGAGGAGAGCCAGAGA-3' and 5'-TTGTGACCTTGCTTGGATGA-3'. Anti-DIG-AP antibody (11093274910; Sigma-Aldrich) and BM Purple reagent (11442074001; Roche) were used to produce a signal in the WISH of vertebrate embryos.

For *Hnrnpa1* (**Figure 3**), at each stage (E7, E7.5, E8, E8.25, E9.5) 3 wild-type embryos from 3 C57BL/6N litters were collected. For *Nkx2.5* and *Isl1*, embryos were collected from crosses between male and female *Hnrnpa1*$^{+/ct}$ mice. All female mice used here were around 3 months old. For *Nkx2.5* (**Figure 5**), 3 groups of embryos from 3 litters were collected at E8, E8.5 and E9.5, respectively. For *Isl1* (**Figure

**6**), 3 groups of embryos from 3 litters were collected at E8, E8.5 and E9.5, respectively. Each group included one wild-type littermate control and one *Hnrnpa1*$^{ct/ct}$ homozygous mutant. The details described in Figures 3, 5 and 6 were detected in all the groups.

**Quantitative RT-PCR (qRT-PCR)**
The whole embryos, pharyngeal tissues and heart tubes were dissected at E9.5 respectively and immediately frozen using liquid nitrogen. Then, the samples were stored at -80 ℃ until they were genotyped. The total RNA was isolated with TRIzol reagent (15596-026; Thermo Fisher Scientific) and standard protocols. First strand cDNA was synthesized using the SuperScript™ III first-strand synthesis system kit (18080-051; Thermo Fisher Scientific). Then, qRT-PCR was performed using TaKaRa SYBR Premix Ex Taq™ kit (RR420W; TaKaRa). For *Hnrnpa1*, six wild-type embryos, nine heterozygous, and eight homozygous mutants were used. Pharyngeal tissues (SHF) and heart tubes were isolated for analyses of SHF and heart tube specific genes (**Supplemental Figure 7**). For analysis of the SHF, eight wild-type littermate controls, five *Hnrnpa1*$^{+/ct}$ heterozygotes and nine *Hnrnpa1*$^{ct/ct}$ homozygotes were used for *Fgf8*, *Fgf10*, *Isl1*, *Mef2c* and *Tbx1*; eight wild-type littermate controls, five *Hnrnpa1*$^{+/ct}$ heterozygotes and eight *Hnrnpa1*$^{ct/ct}$ homozygotes were used for *Nkx2.5*; eight wild-type littermate controls, six *Hnrnpa1*$^{+/ct}$ heterozygotes and nine *Hnrnpa1*$^{ct/ct}$ homozygotes were used for *Acvr1*, *Bmpr1a* and *Jag1*. For analysis of the heart tube, eight wild-type embryos, eight heterozygous, and nine homozygous mutants were dissected for analysis of *Mlc2a*, *Mlc2v* and *Nkx2.5*; eleven wild-type embryos, nine heterozygous, and nine homozygous mutants were dissected for analysis of *Mef2c*; eleven wild-type embryos, eleven heterozygous, and nine homozygous mutants were dissected for analysis of *Myocd* and *SRF*. *GAPDH* was used as an internal control (9). qRT-PCR primers for *Hnrnpa1*(designed), *Acvr1* (10), *Bmpr1a*, *Jag1*, *Myocd* (11), *Fgf8* (12), *Fgf10*, *Mef2c*, *Tbx1* (13), *Isl1* (14), *Mlc2a*, *Mlc2v* (15), *Nkx2.5* (designed) and *SRF* (16) are listed in **Supplemental Table 6**.

**Western blot analysis**
Embryos were dissected at E9.5 and lysed with ice-cold radioimmunoprecipitation assay (RIPA) buffer (50 mM Tris-HCl, pH 8.0, 150 mM NaCl, 0.1% SDS, 0.5% sodium deoxycholate, 0.1% Triton-X-100) supplemented with protease and phosphatase inhibitors (Roche). The embryo lysates were then passed through 1 mL syringes with needles for around 20 times. The supernatant was obtained after centrifugation at 12,000 *g* for 10 min at 4 ℃. Extracted protein samples were denatured in 1 × Laemmli buffer and subjected to SDS-PAGE. Total proteins were then transferred onto nitrocellulose membrane (GE Healthcare). The membrane was incubated with primary antibodies overnight at 4 ℃ after 1h blockage in BSA at room temperature. β-Actin was used as the loading control. Primary antibodies used: anti-mouse hnRNP A1 (sc-32301; Santa Cruz); anti-β-Actin (A2228; Sigma-Aldrich). The blots were detected using chemiluminescence.

***In vitro* differentiation of mESCs into cardiomyocytes**
**CRISPR knockout of *Hnrnpa1***
CRISPR/Cas9 was used to generate *Hnrnpa1* knockout Nkx2.5-EGFP-mESCs (17). Briefly, sgRNA (GGTCGTGGAGGCGGTTTTGG) was linked into the Cas9 plasmid (48139; Addgene) for lipofetamine 2000 (Life tech) induced transfection. Then, cells were cultured under fresh mESC medium until the colonies emerged after the three days of puromycin (Life Tech, 2.5 μg/mL) treatment. Two *Hnrnpa1*$^{-/-}$ cell lines (KO1 and KO2) were utilized for further experiments, which were homozygous through genotype analysis. PCR and Sanger sequencing were performed to confirm the sequences in the knockout region and predicted off-target genomic loci (**Supplemental Table 7**).

**Cardiomyocyte differentiation:**
mESCs were cultured and expanded with mESCs maintenance medium (15% FBS, 85% DMEM, L-Glutamine, NEAA, 0.1 mM 2-mercaptoethanol, and 10 ng/mL LIF), which was changed on a daily basis. When cell colonies cultured on fibronectin-coated plate reached about 80% confluence, the medium

was removed. After washing by PBS, the cells were digested with 0.05% trypsin-EDTA for 3 minutes at 37 ℃. After centrifugation, the cells were suspended in the differentiation medium (15% FBS, 85% DMEM, L-Glutamine, and NEAA). After determining the cell number, the cells were seeded at a density of $2 \times 10^5$/mL into 6-well ultra-low attachment plates (2 mL/well). Cells were cultured for 2 days to allow their spontaneous aggregation. Differentiation medium was changed every day to allow growth of the EBs. At the end of day 6, EBs were transferred to a fibronectin-coated plate to allow them to adhere to the plate for further differentiation. The medium was changed every day until the end of the observation period (day 9).

**Flow cytometry analysis of Nkx2.5-EGFP positive percentage during differentiation**

During the differentiation of Nkx2.5-EGFP-mESCs, GFP was expressed when Nkx2.5 was expressed. To determine the percentage of Nkx2.5-EGFP cells during differentiation, the cells were collected and flow cytometry analysis was performed at day 7. Differentiated mESCs in each group were trypsinized with 0.05% trypsin for 5 minutes and then collected through centrifugation, respectively. Cells were washed with 1 x PBS, followed by fixation with 4% paraformaldehyde (PFA). The cells were kept in dark, in 4 ℃ refrigerator until they were analyzed. Data were analyzed by collecting 50000 events on Beckman Coulter FC500 using CXP Analysis 2.0 software. For each cell type, *in vitro* differentiation was performed three times. Cell samples for flow cytometry analysis were collected at day 7 of the differentiation period. Undifferentiated wild-type mESCs were collected as control.

**References**


1. Sobel E, Sengul H, and Weeks DE. Multipoint estimation of identity-by-descent probabilities at arbitrary positions among marker loci on general pedigrees. *Human heredity*. 2001;52(3):121-31.
2. Li H, and Durbin R. Fast and accurate short read alignment with Burrows-Wheeler transform. *Bioinformatics*. 2009;25(14):1754-60.
3. Li H, Handsaker B, Wysoker A, Fennell T, Ruan J, Homer N, Marth G, Abecasis G, Durbin R, and Genome Project Data Processing S. The Sequence Alignment/Map format and SAMtools. *Bioinformatics*. 2009;25(16):2078-9.
4. Wang K, Li M, and Hakonarson H. ANNOVAR: functional annotation of genetic variants from high-throughput sequencing data. *Nucleic acids research*. 2010;38(16):e164. doi: 10.1093/nar/gkq603.
5. Sharpe J, Ahlgren U, Perry P, Hill B, Ross A, Hecksher-Sorensen J, Baldock R, and Davidson D. Optical projection tomography as a tool for 3D microscopy and gene expression studies. *Science*. 2002;296(5567):541-5.
6. Cai CL, Liang X, Shi Y, Chu PH, Pfaff SL, Chen J, and Evans S. Isl1 identifies a cardiac progenitor population that proliferates prior to differentiation and contributes a majority of cells to the heart. *Developmental cell*. 2003;5(6):877-89.
7. Prall OW, Menon MK, Solloway MJ, Watanabe Y, Zaffran S, Bajolle F, Biben C, McBride JJ, Robertson BR, Chaulet H, et al. An Nkx2-5/Bmp2/Smad1 negative feedback loop controls heart progenitor specification and proliferation. *Cell*. 2007;128(5):947-59.
8. Kang J, Nathan E, Xu SM, Tzahor E, and Black BL. Isl1 is a direct transcriptional target of Forkhead transcription factors in second-heart-field-derived mesoderm. *Developmental biology*. 2009;334(2):513-22.
9. Yin M, Zhang L, Sun XM, Mao LF, and Pan J. Lack of apoE causes alteration of cytokines expression in young mice liver. *Molecular biology reports*. 2010;37(4):2049-54.
10. Zhou X, Wang Y, Ongaro L, Boehm U, Kaartinen V, Mishina Y, and Bernard DJ. Normal gonadotropin production and fertility in gonadotrope-specific Bmpr1a knockout mice. *The Journal of endocrinology*. 2016;229(3):331-41.
11. Lopes M, Goupille O, Saint Cloment C, Lallemand Y, Cumano A, and Robert B. Msx genes define a population of mural cell precursors required for head blood vessel maturation. *Development*. 2011;138(14):3055-66.
12. Ryckebusch L, Bertrand N, Mesbah K, Bajolle F, Niederreither K, Kelly RG, and Zaffran S. Decreased levels of embryonic retinoic acid synthesis accelerate recovery from arterial growth delay in a mouse model of DiGeorge syndrome. *Circulation research*. 2010;106(4):686-94.
13. High FA, Jain R, Stoller JZ, Antonucci NB, Lu MM, Loomes KM, Kaestner KH, Pear WS, and Epstein JA. Murine Jagged1/Notch signaling in the second heart field orchestrates Fgf8 expression and tissue-tissue interactions during outflow tract development. *The Journal of clinical investigation*. 2009;119(7):1986-96.
14. Zhang Q, Yang Z, Jia Z, Liu C, Guo C, Lu H, Chen P, Ma K, Wang W, and Zhou C. ISL-1 is overexpressed in non-Hodgkin lymphoma and promotes lymphoma cell proliferation by forming a p-STAT3/p-c-Jun/ISL-1 complex. *Molecular cancer*. 2014;13:181. doi: 10.1186/1476-4598-13-181.
15. Koren L, Elhanani O, Kehat I, Hai T, and Aronheim A. Adult cardiac expression of the activating transcription factor 3, ATF3, promotes ventricular hypertrophy. *PloS one*. 2013;8(7):e68396. doi: 10.1371/journal.pone.0068396.
16. Kawakami-Schulz SV, Verdoni AM, Sattler SG, Jessen E, Kao WW, Ikeda A, and Ikeda S. Serum response factor: positive and negative regulation of an epithelial gene expression network in the destrin mutant cornea. *Physiological genomics*. 2014;46(8):277-89.
17. Cong L, Ran FA, Cox D, Lin S, Barretto R, Habib N, Hsu PD, Wu X, Jiang W, Marraffini LA, et al. Multiplex genome engineering using CRISPR/Cas systems. *Science*. 2013;339(6121):819-23.

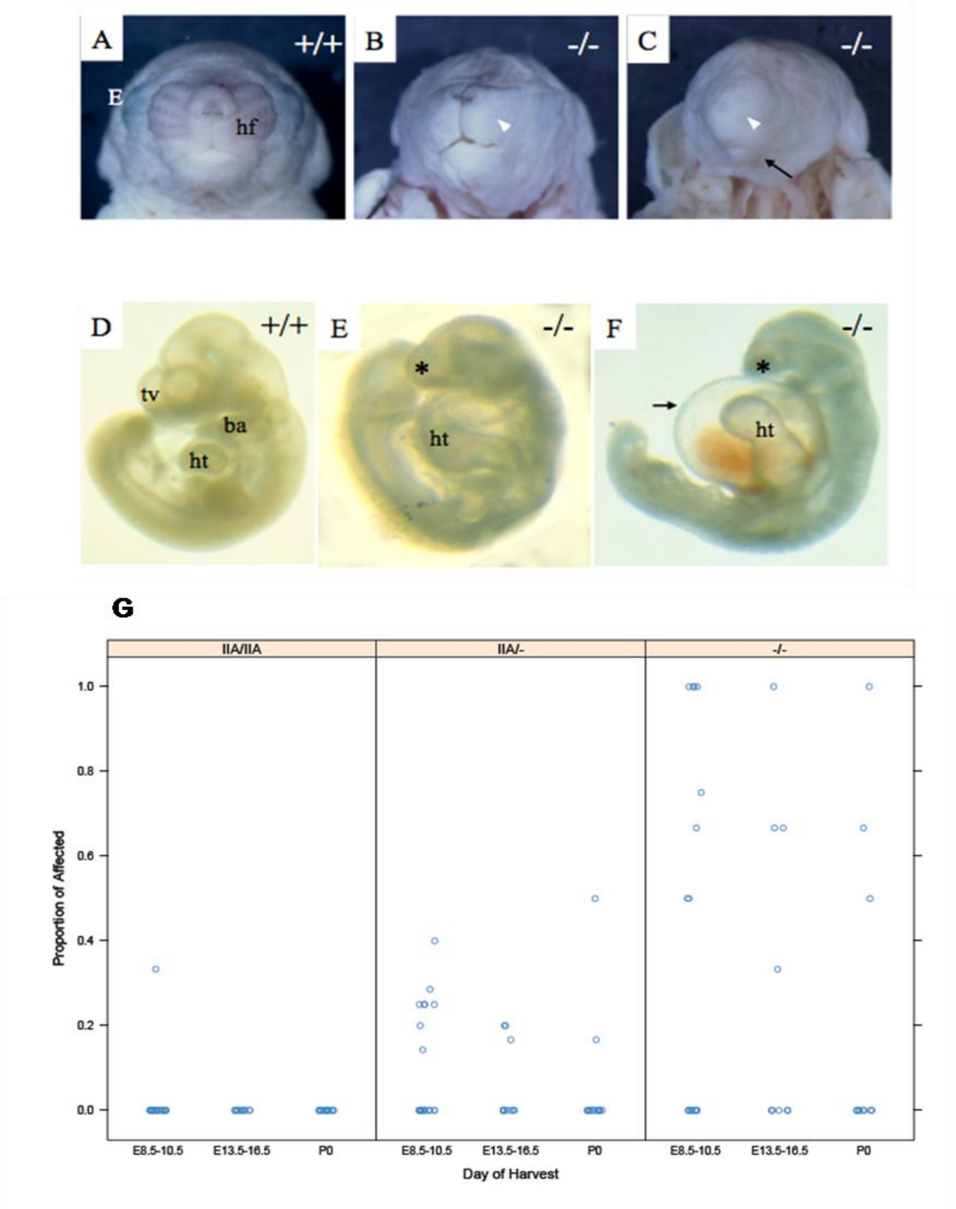


**Supplemental Figure 1**

**Incomplete penetrance of the CHD phenotypes at different stages from *IIA*$^{+/-}$ x *IIA*$^{+/-}$ crosses with hybrid genetic background**

Phenotypes of *IIA*$^{-/-}$ newborns and embryos. **(A-C)** Wild-type and *IIA*$^{-/-}$ newborn mice at P1; no hair follicles around the frontal nasal area, minimal or no nasal structures and small or no eyes were observed in *IIA*$^{-/-}$ newborns. **(D-F)** Wild-type and *IIA*$^{-/-}$ embryos at late E9.5; *IIA*$^{-/-}$ embryos displayed incomplete heart looping, cardiac edema, small telencephalic vesicles (asterisks marking the prospective telencephalic vesicles) which were too close together, and reduced branchial arches. ba-branchial arch, E-eye, hf-hair follicles, ht-heart-tube, tv-telencephalic vesicles. **(G)** The figure shows the proportion of heart defects for each litters collected from *IIA*$^{+/-}$ x *IIA*$^{+/-}$ crosses with different *IIA* genotypes. The mean of the proportion of heart defects for *IIA*$^{+/+}$, *IIA*$^{+/-}$ and *IIA*$^{-/-}$ are (0.74%, SD: 3.4%), (6.80%, SD: 3.3%) and (30.4%, SD: 3.7%) respectively. It shows that heart defect mutants with *IIA*$^{-/-}$ genotype have a significant increase with $p = 7.4 \times 10^{-8}$ from ANOVA test. However, it was also observed a great variance in the proportion of heart defects in the *IIA*$^{-/-}$ group.

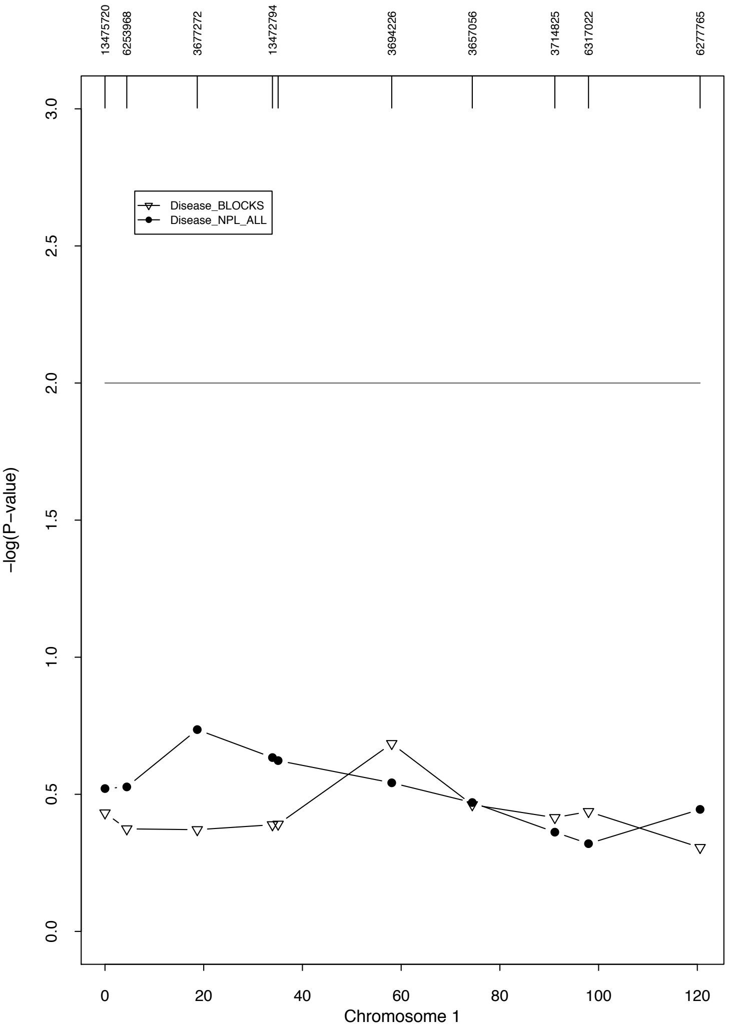
13475720
6253968
3677272
13472794
3694226
3657056
3714825
6317022
6277765
Disease_BLOCKS
Disease_NPL_ALL
−log(P−value)
Chromosome 1

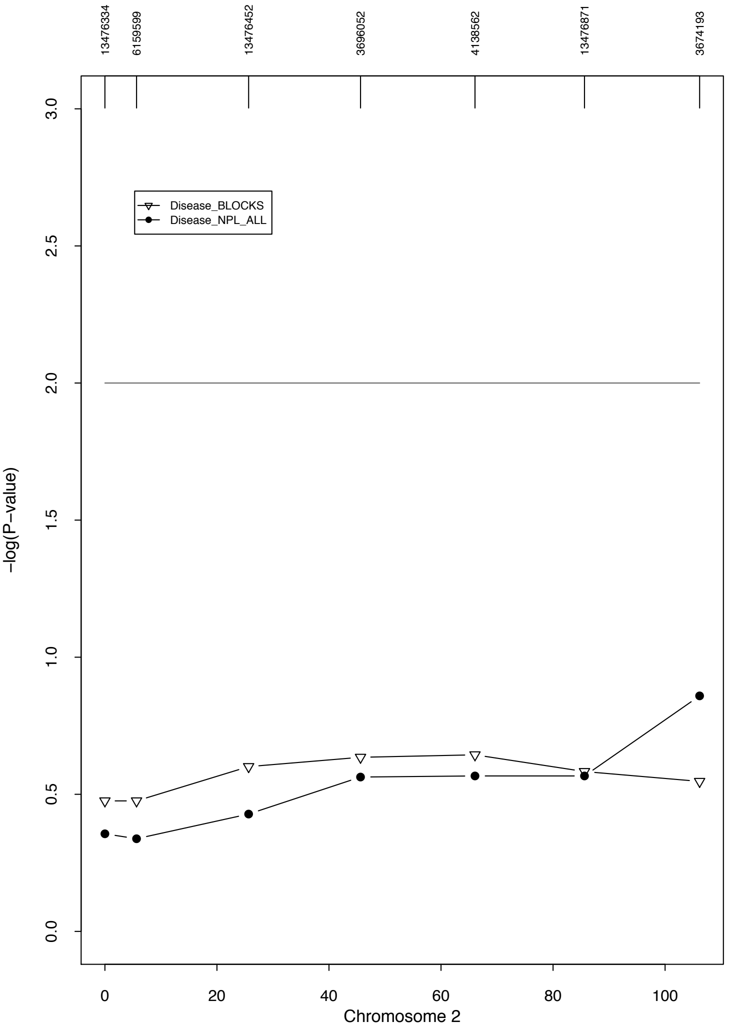
13476334
6159599
13476452
3696052
4138562
13476871
3674193
Disease_BLOCKS
Disease_NPL_ALL
−log(P−value)
Chromosome 2

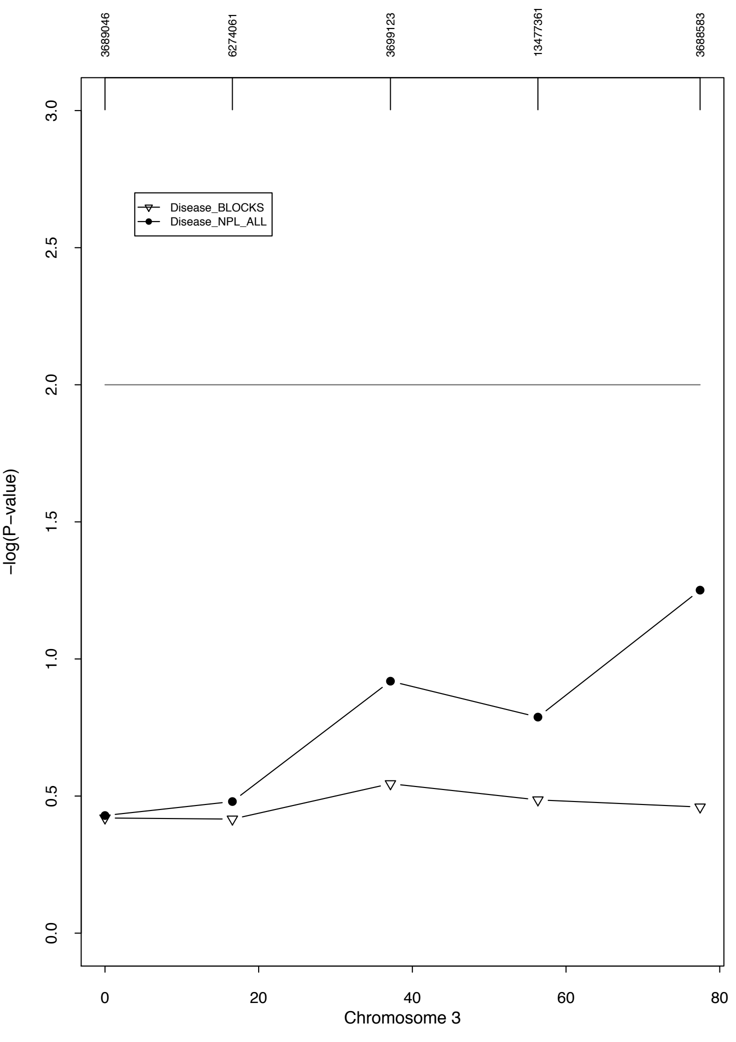
3689046
6274061
3699123
13477361
3688583
Disease_BLOCKS
Disease_NPL_ALL
−log(P−value)
Chromosome 3

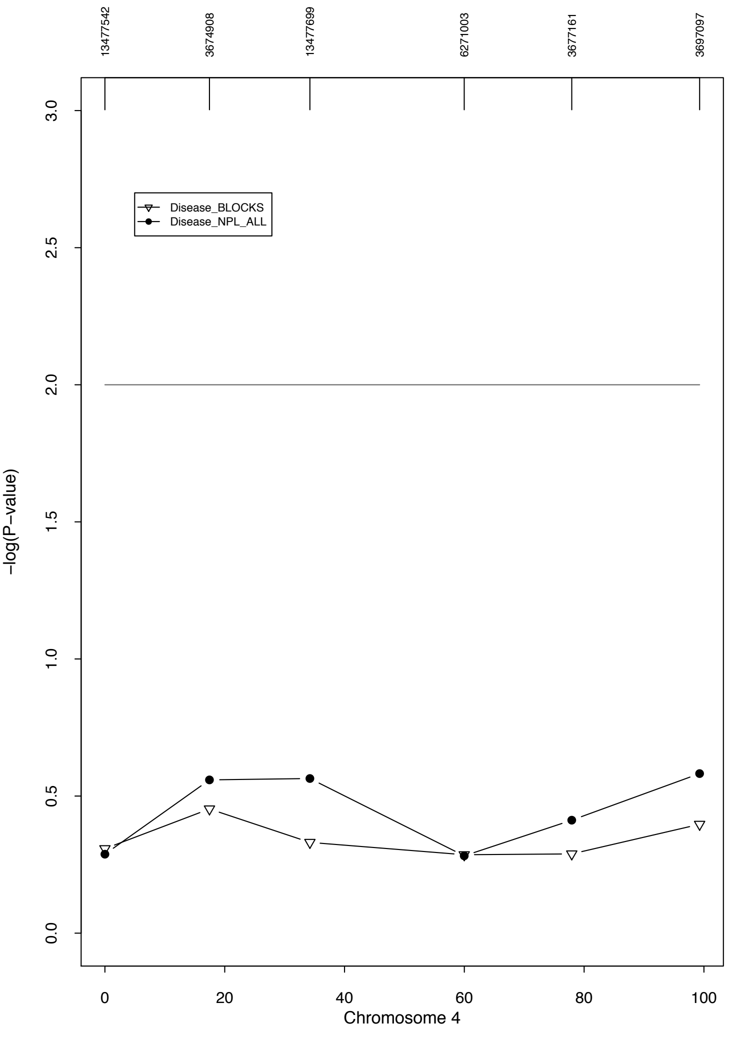
13477542
3674908
13477699
6271003
3677161
3697097
Disease_BLOCKS
Disease_NPL_ALL
−log(P−value)
Chromosome 4

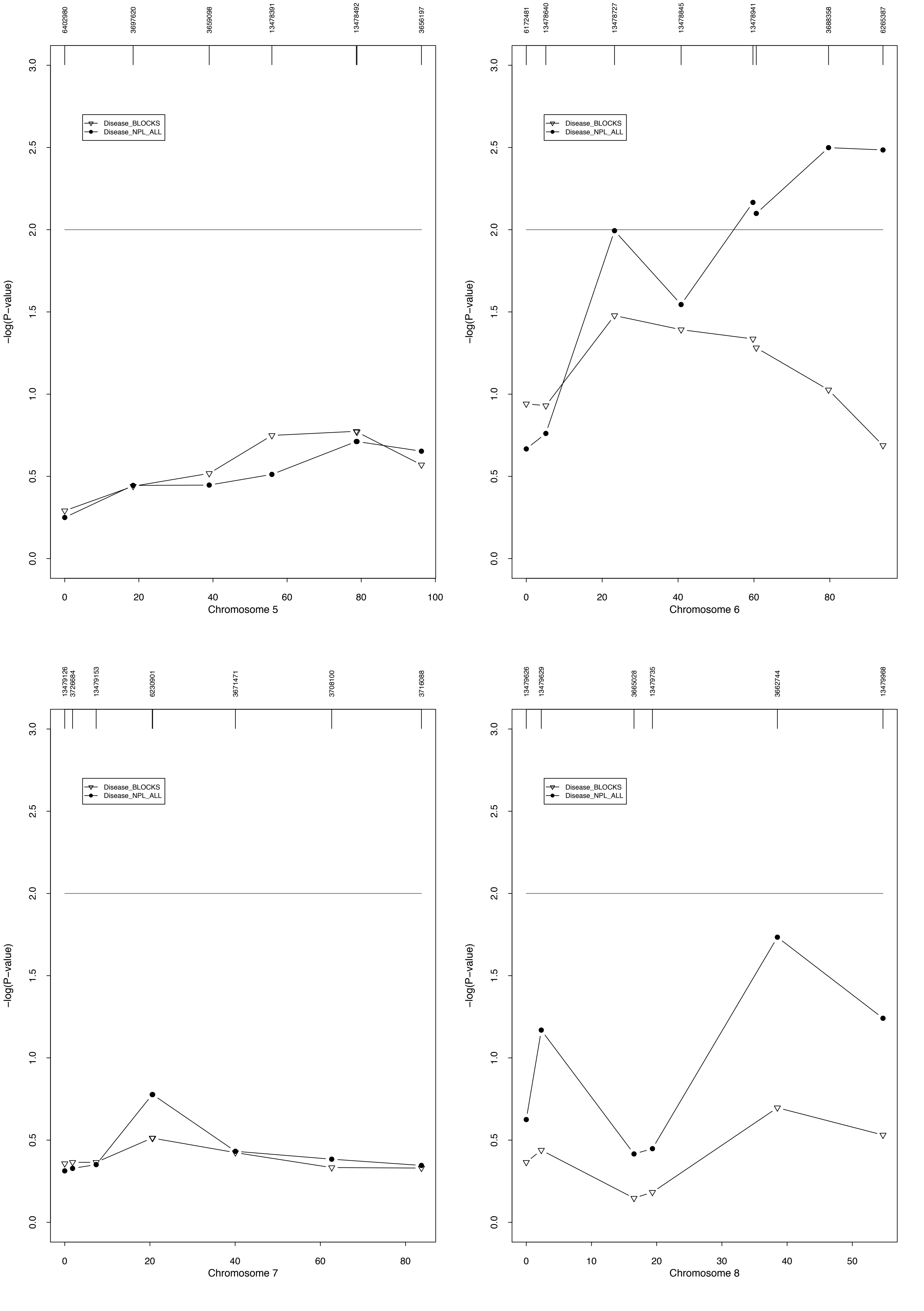
6402980
3697620
3659098
13478391
13478492
3656197
Disease_BLOCKS
Disease_NPL_ALL
-log(P-value)
Chromosome 5
6172481
13478640
13478727
13478845
13478941
3688358
6265387
Disease_BLOCKS
Disease_NPL_ALL
-log(P-value)
Chromosome 6
13479126
3726684
13479153
6230901
3671471
3708100
3716088
Disease_BLOCKS
Disease_NPL_ALL
-log(P-value)
Chromosome 7
13479626
13479629
3665028
13479735
3662744
13479968
Disease_BLOCKS
Disease_NPL_ALL
-log(P-value)
Chromosome 8

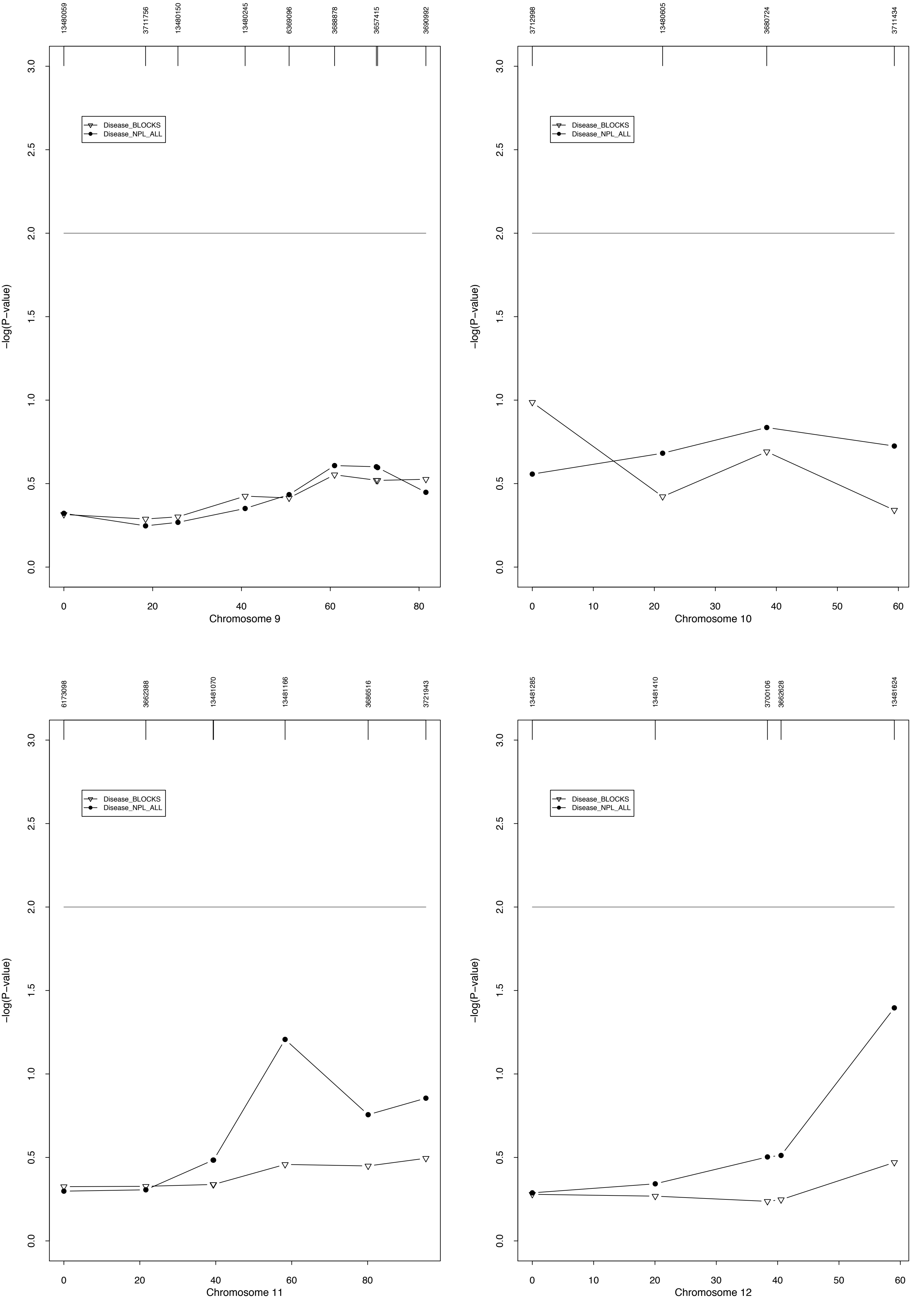
13480059
3711756
13480150
13480245
6369096
3688878
3657415
3690992
Disease_BLOCKS
Disease_NPL_ALL
-log(P-value)
Chromosome 9
3712998
13480605
3680724
3711434
Disease_BLOCKS
Disease_NPL_ALL
-log(P-value)
Chromosome 10
6173098
3662388
13481070
13481166
3686516
3721943
Disease_BLOCKS
Disease_NPL_ALL
-log(P-value)
Chromosome 11
13481285
13481410
3700106
3662628
13481624
Disease_BLOCKS
Disease_NPL_ALL
-log(P-value)
Chromosome 12

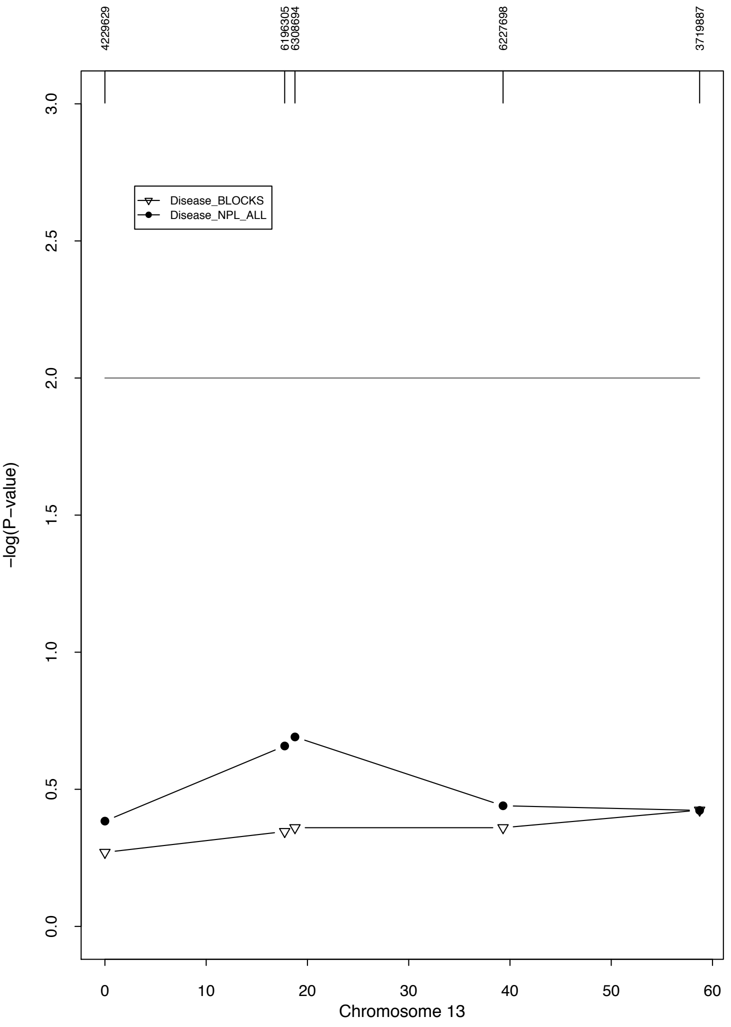
4229629
6196305
6308694
6227698
3719887
Disease_BLOCKS
Disease_NPL_ALL
-log(P-value)
Chromosome 13

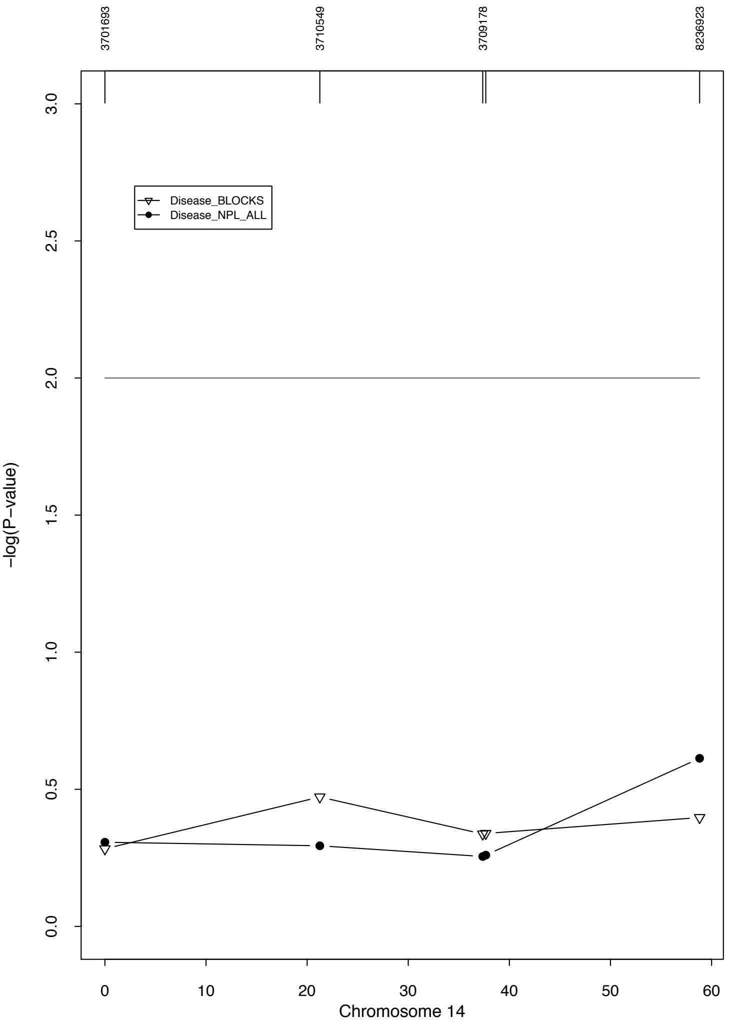
3701693
3710549
3709178
8236923
Disease_BLOCKS
Disease_NPL_ALL
-log(P-value)
Chromosome 14

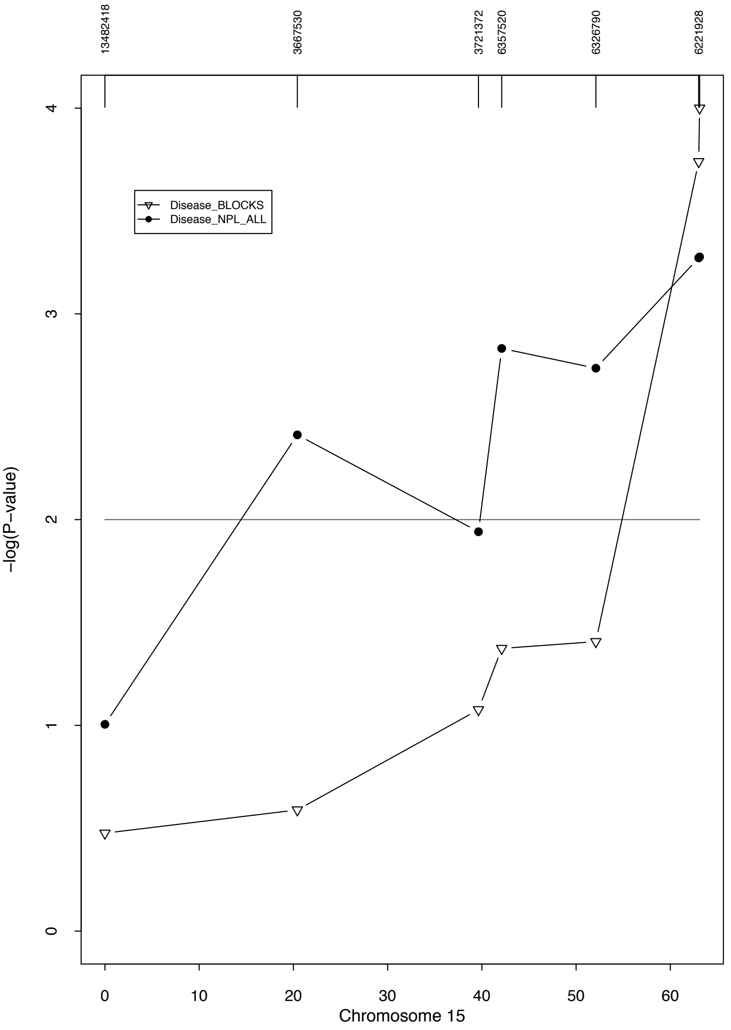
13482418
3667530
3721372
6357520
6326790
6221928
Disease_BLOCKS
Disease_NPL_ALL
-log(P-value)
Chromosome 15

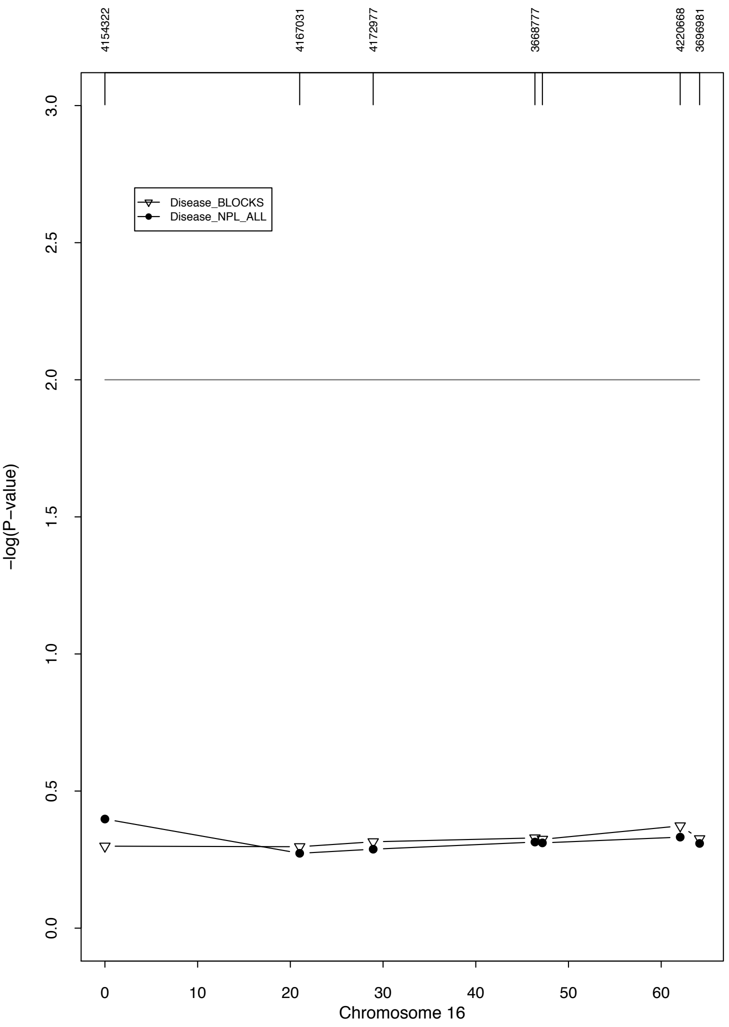
4154322
4167031
4172977
3668777
4220668
3696981
Disease_BLOCKS
Disease_NPL_ALL
-log(P-value)
Chromosome 16

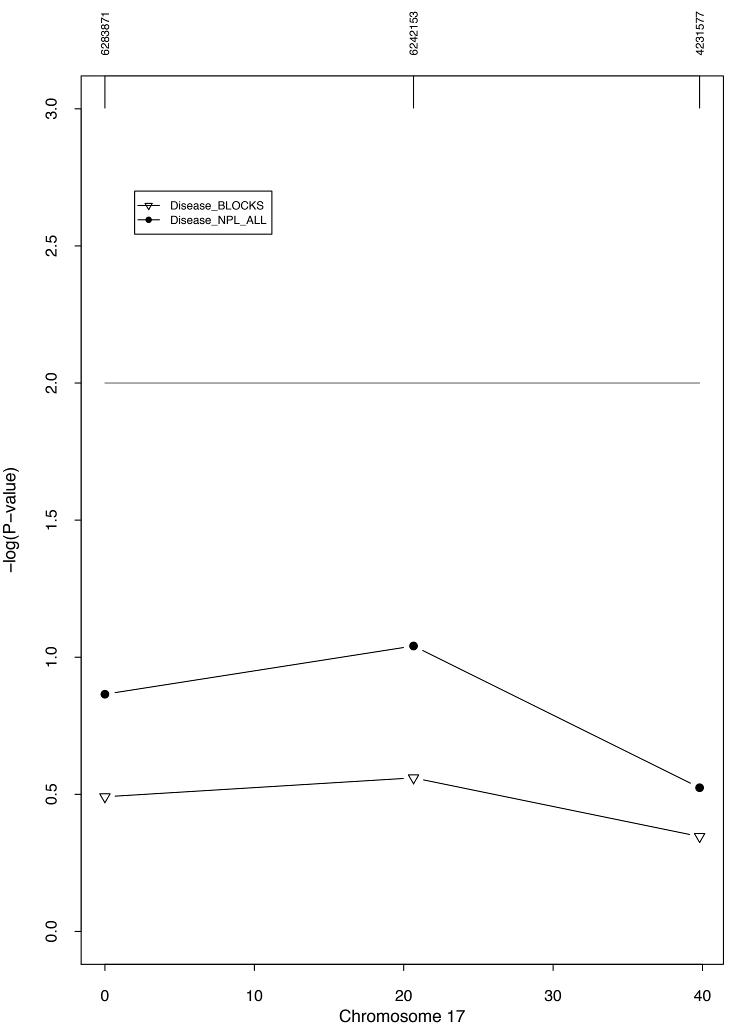
6283871
6242153
4231577
Disease_BLOCKS
Disease_NPL_ALL
-log(P-value)
Chromosome 17

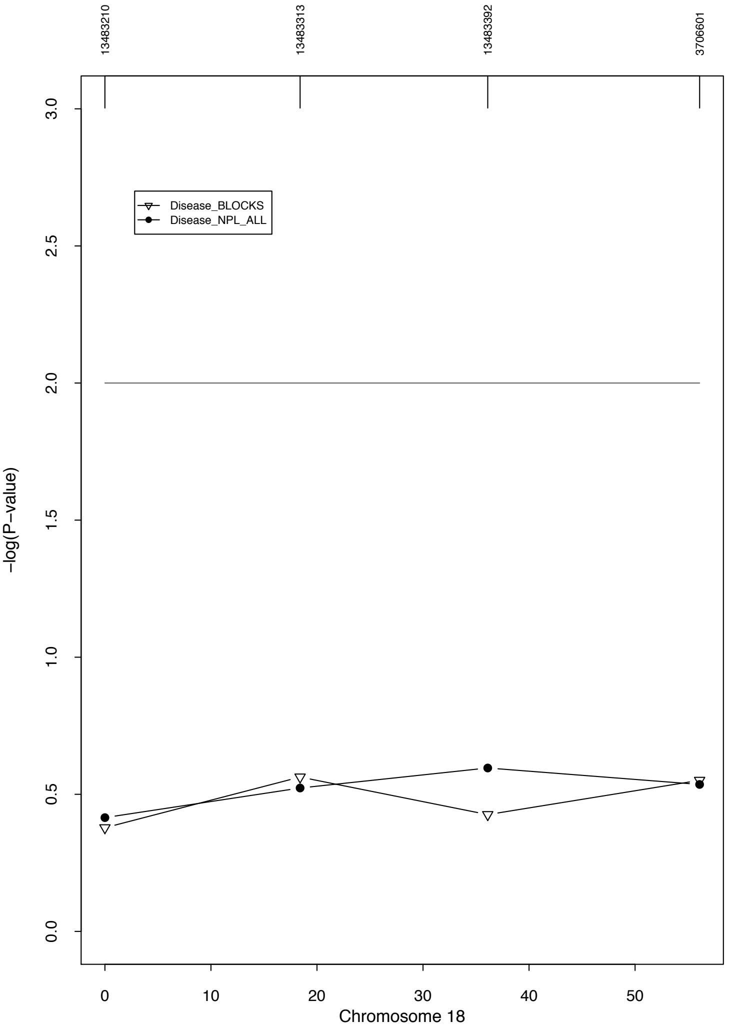
13483210
13483313
13483392
3706601
Disease_BLOCKS
Disease_NPL_ALL
-log(P-value)
Chromosome 18

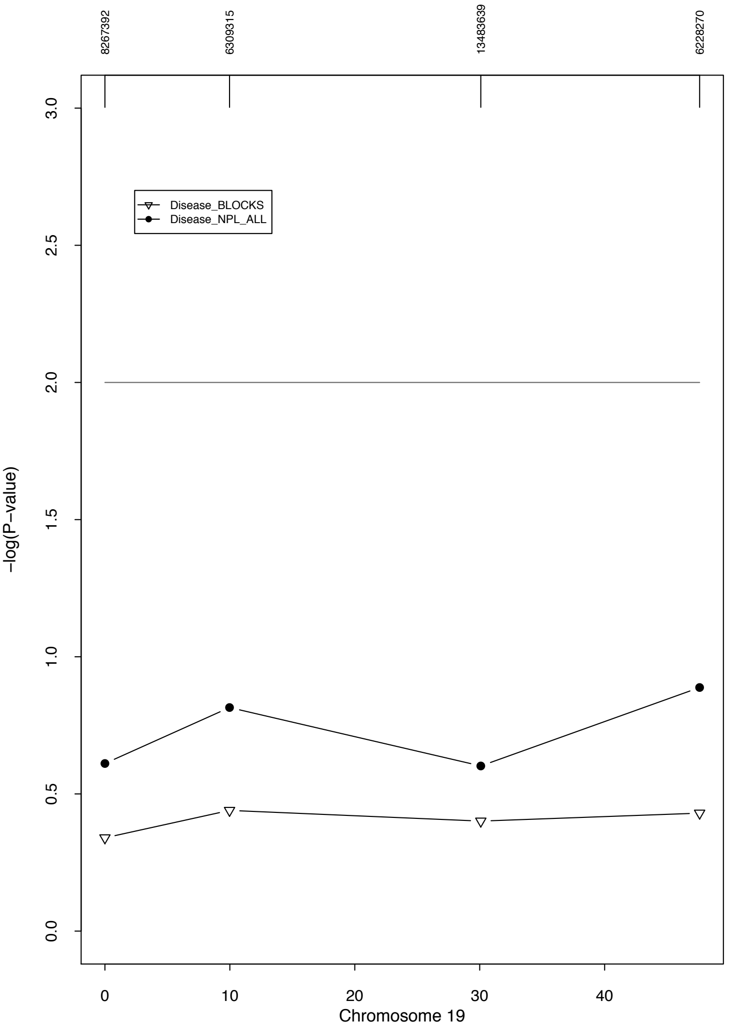
8267392
6309315
13483639
6228270
Disease_BLOCKS
Disease_NPL_ALL
-log(P-value)
Chromosome 19

**Supplemental Figure 2**

**Genome-wide linkage analysis of lethal at birth**

The *X*-axis represents the relative location of markers on a chromosome and the *Y*-axis represents the –log(p) for NPL-ALL scores and BLOCK recessive scores.

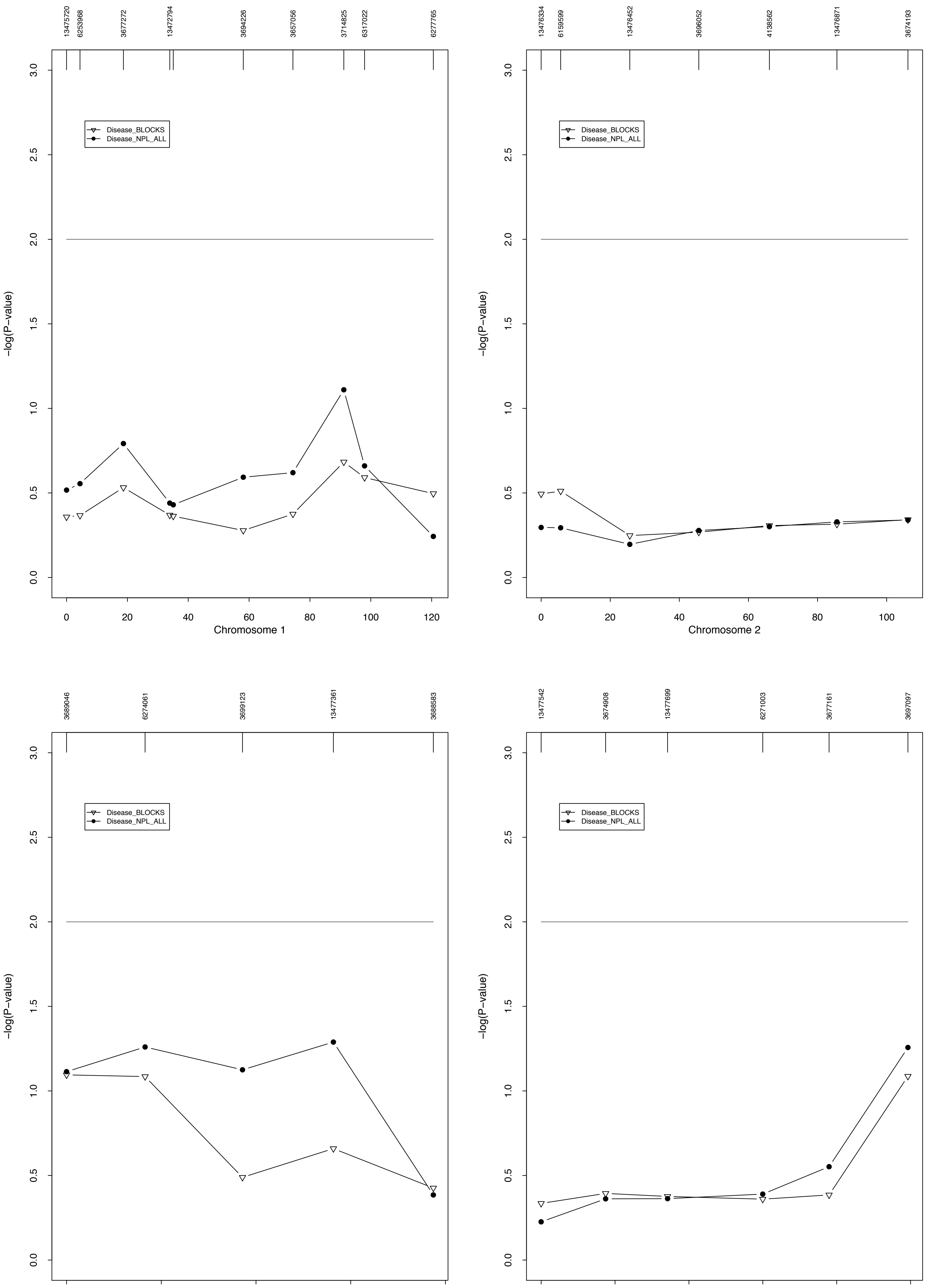

13475720
6253968
3677272
13472794
3694226
3657056
3714825
6317022
6277765
Disease_BLOCKS
Disease_NPL_ALL
-log(P-value)
Chromosome 1
13476334
6159599
13476452
3696052
4138562
13476871
3674193
Disease_BLOCKS
Disease_NPL_ALL
-log(P-value)
Chromosome 2
3689046
6274061
3699123
13477361
3688583
Disease_BLOCKS
Disease_NPL_ALL
-log(P-value)
Chromosome 3
13477542
3674908
13477699
6271003
3677161
3697097
Disease_BLOCKS
Disease_NPL_ALL
-log(P-value)
Chromosome 4

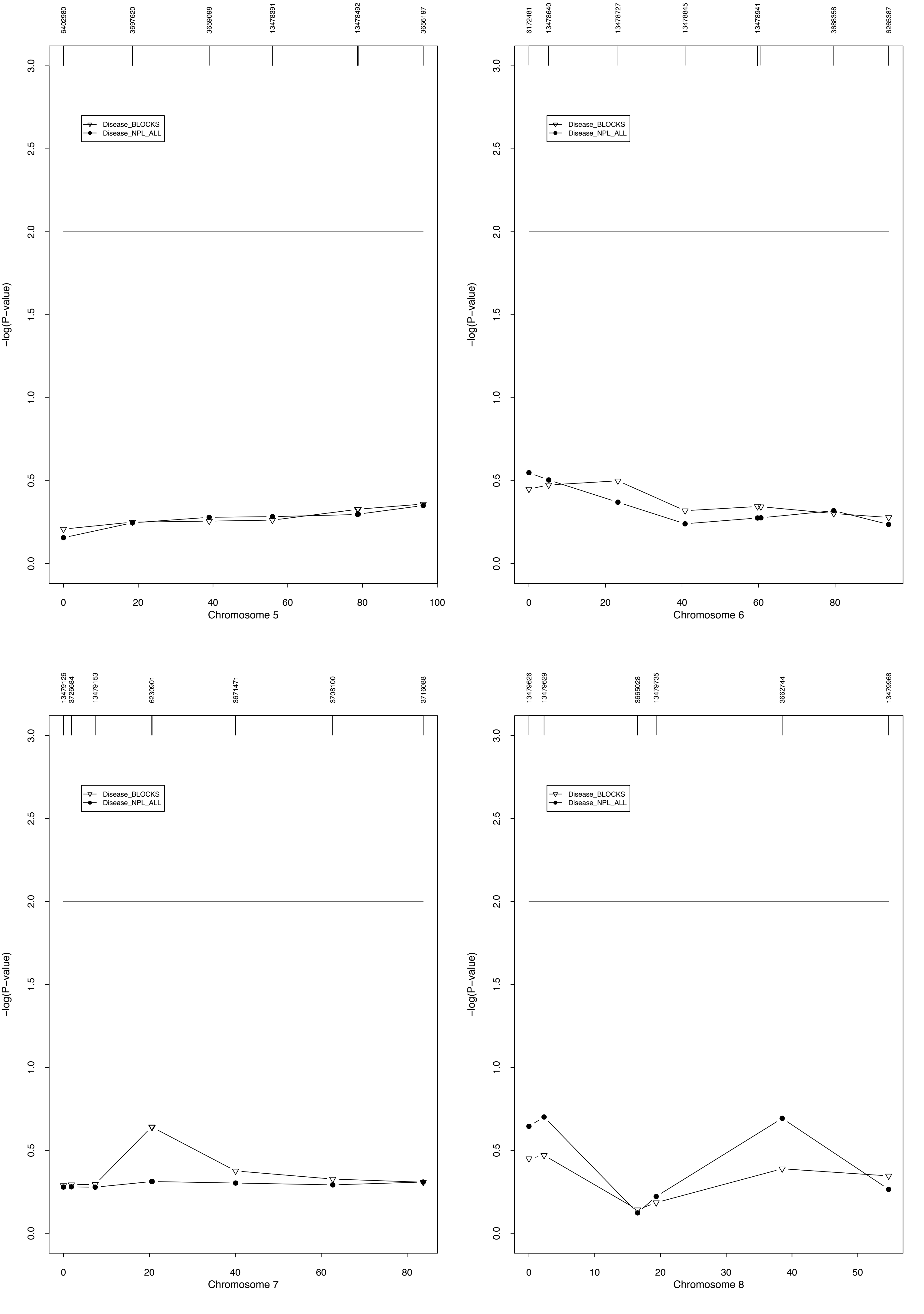

6402980
3697620
3659098
13478391
13478492
3656197
Disease_BLOCKS
Disease_NPL_ALL
-log(P-value)
Chromosome 5
6172481
13478640
13478727
13478845
13478941
3688358
6265387
Disease_BLOCKS
Disease_NPL_ALL
-log(P-value)
Chromosome 6
13479126
3726684
13479153
6230901
3671471
3708100
3716088
Disease_BLOCKS
Disease_NPL_ALL
-log(P-value)
Chromosome 7
13479626
13479629
3665028
13479735
3662744
13479968
Disease_BLOCKS
Disease_NPL_ALL
-log(P-value)
Chromosome 8

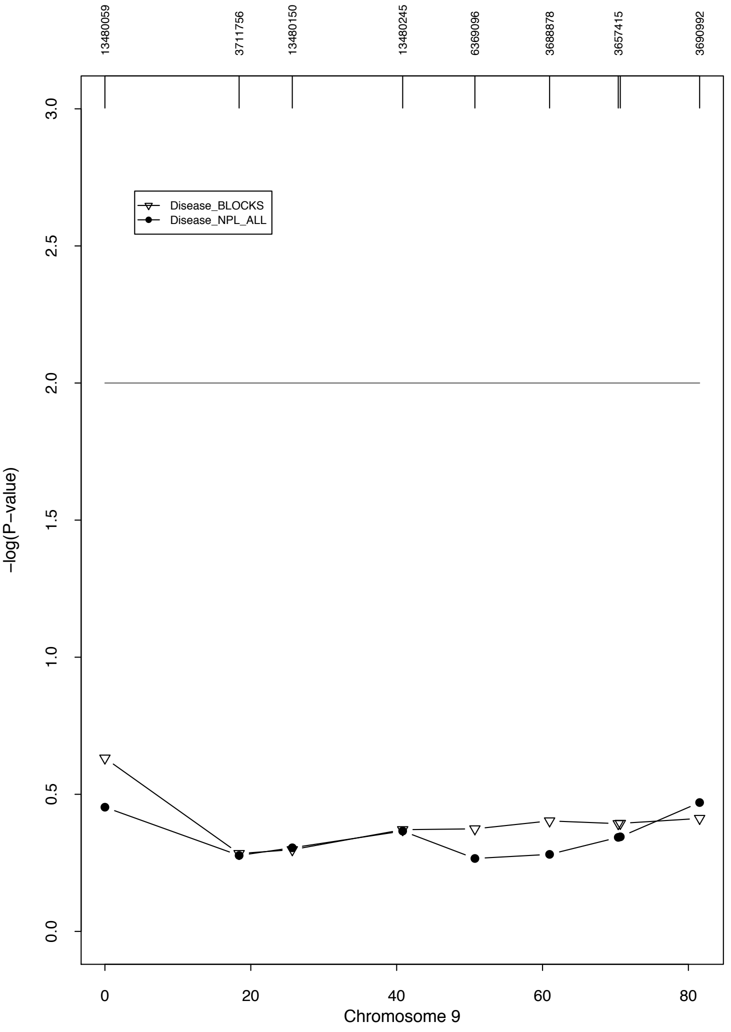
13480059
3711756
13480150
13480245
6369096
3688878
3657415
3690992
Disease_BLOCKS
Disease_NPL_ALL
-log(P-value)
Chromosome 9

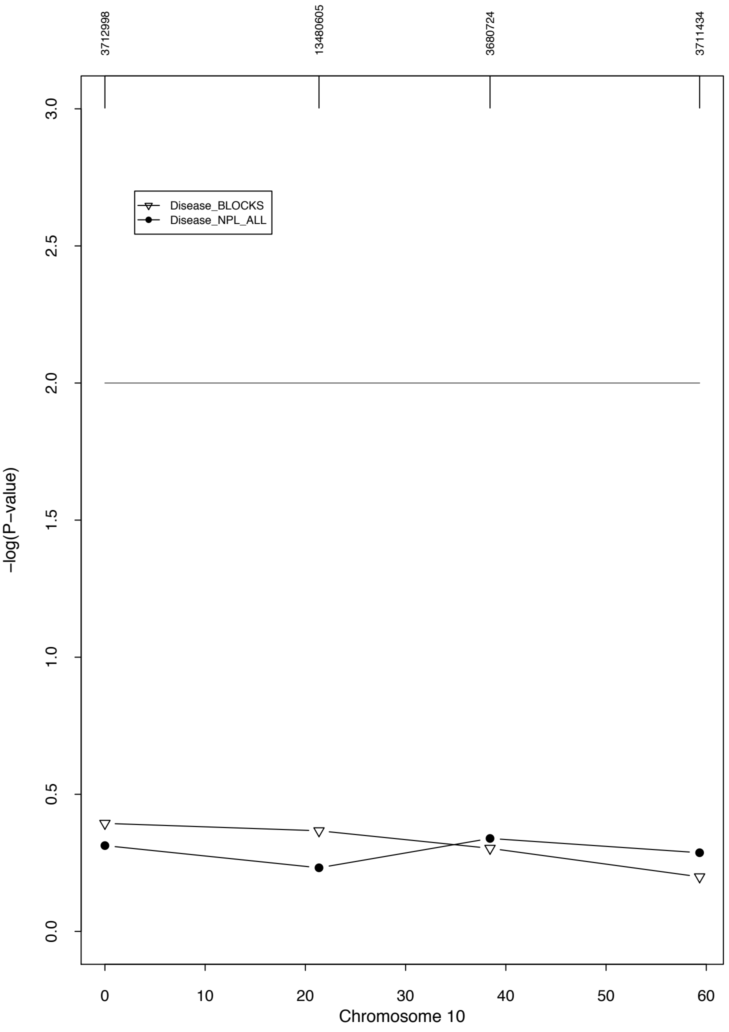
3712998
13480605
3680724
3711434
Disease_BLOCKS
Disease_NPL_ALL
-log(P-value)
Chromosome 10

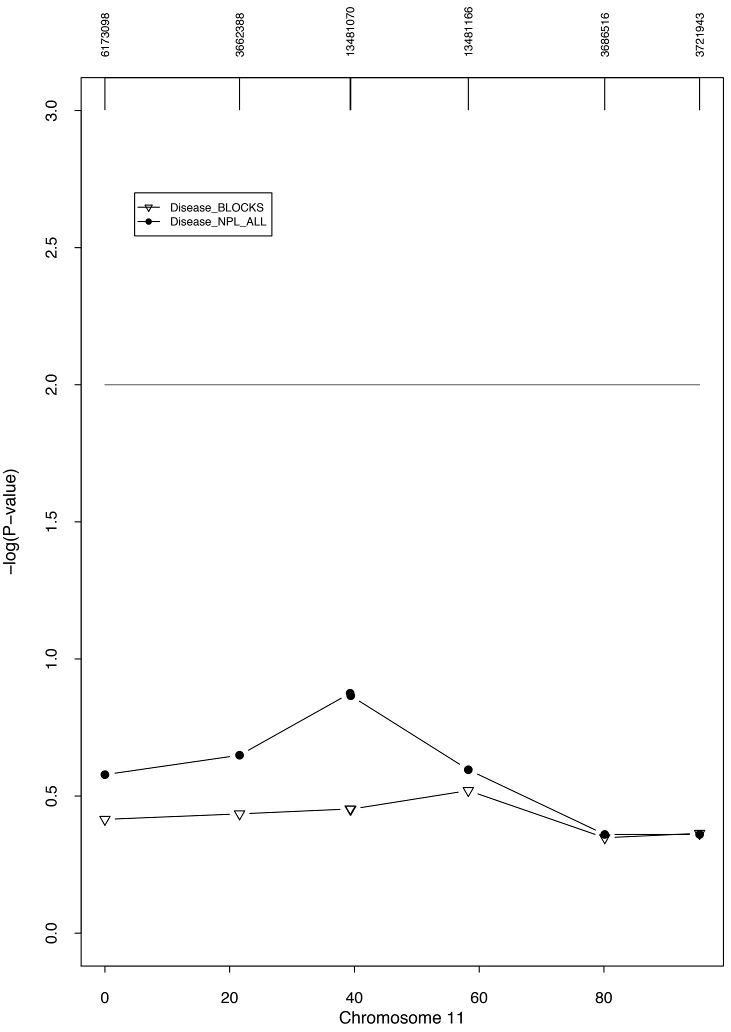
6173098
3662388
13481070
13481166
3686516
3721943
Disease_BLOCKS
Disease_NPL_ALL
-log(P-value)
Chromosome 11

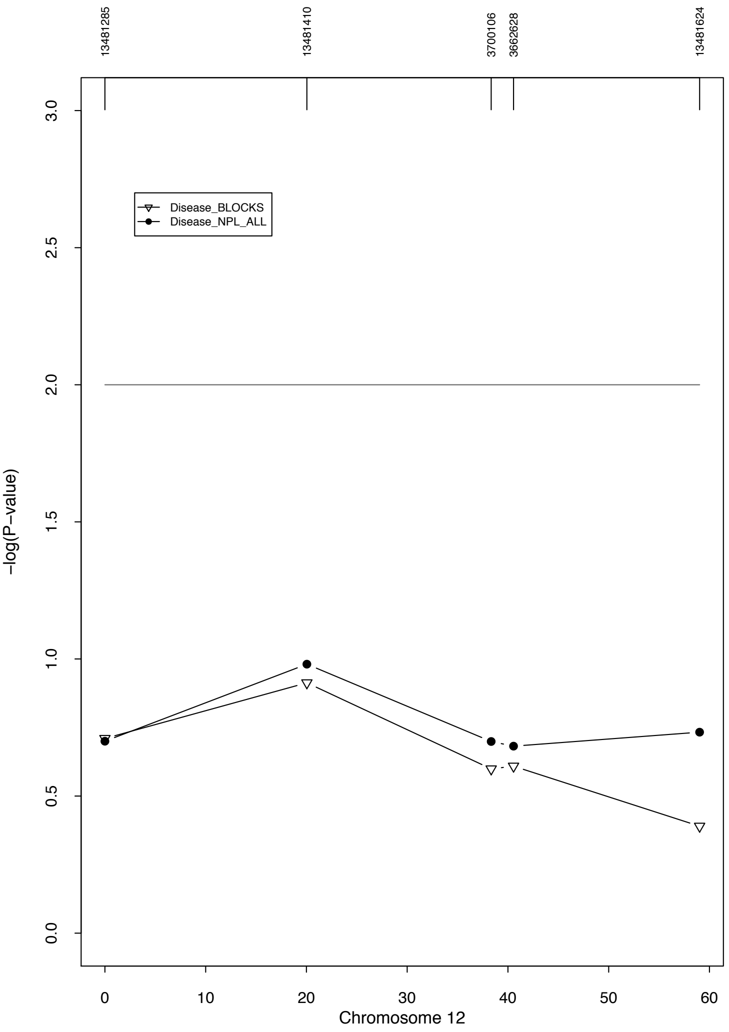
13481285
13481410
3700106
3662628
13481624
Disease_BLOCKS
Disease_NPL_ALL
-log(P-value)
Chromosome 12

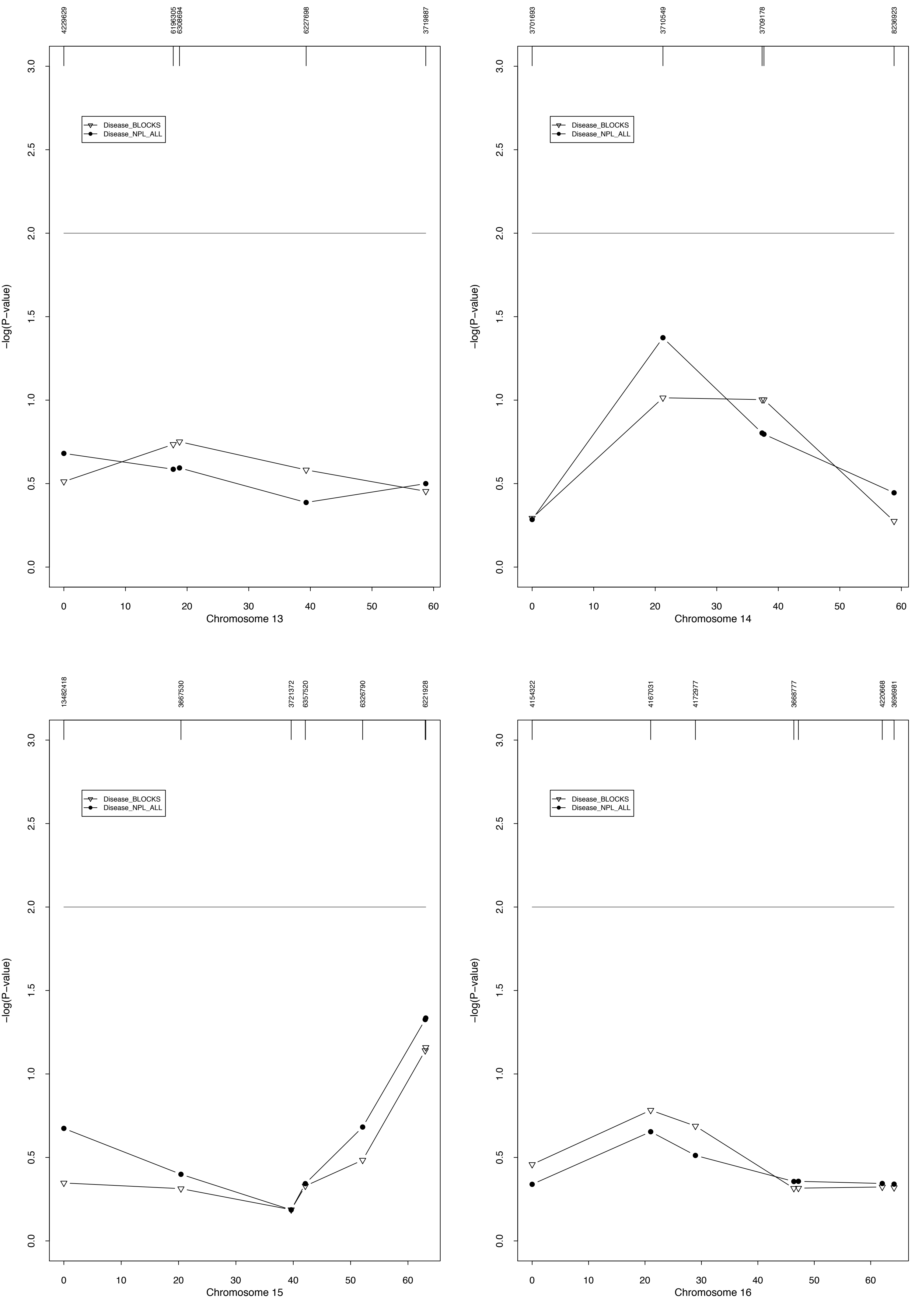
4229629
6196305
6308694
6227698
3719887
Disease_BLOCKS
Disease_NPL_ALL
-log(P-value)
Chromosome 13
3701693
3710549
3709178
8236923
Disease_BLOCKS
Disease_NPL_ALL
-log(P-value)
Chromosome 14
13482418
3667530
3721372
6357520
6326790
6221928
Disease_BLOCKS
Disease_NPL_ALL
-log(P-value)
Chromosome 15
4154322
4167031
4172977
3668777
4220668
3696981
Disease_BLOCKS
Disease_NPL_ALL
-log(P-value)
Chromosome 16

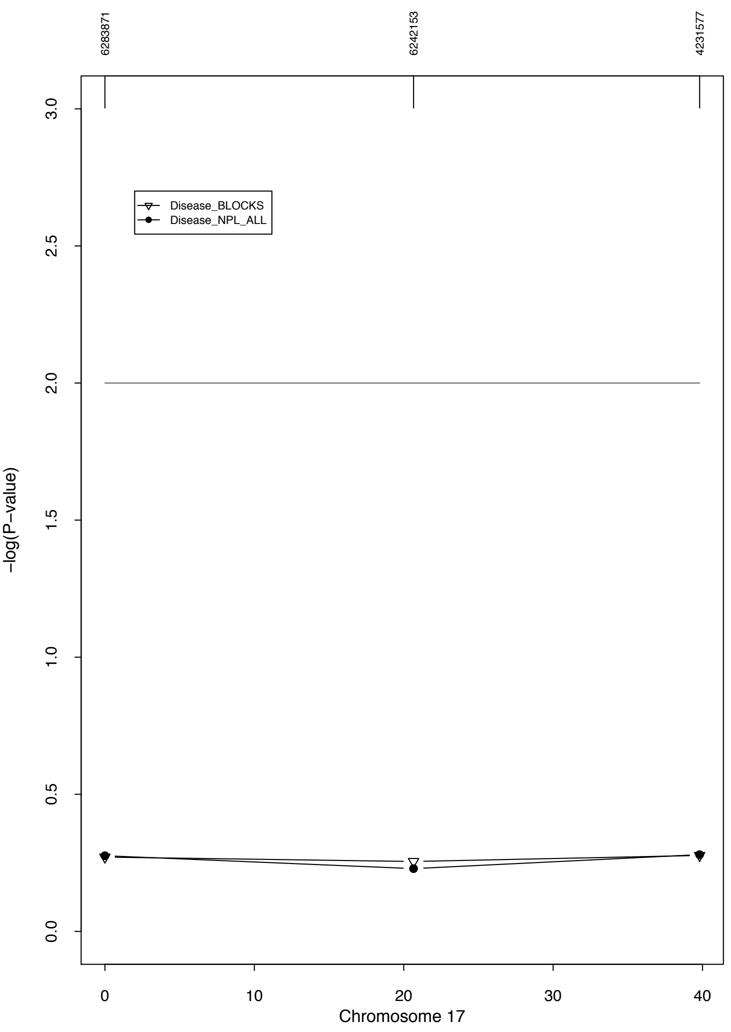
6283871
6242153
4231577
Disease_BLOCKS
Disease_NPL_ALL
−log(P−value)
Chromosome 17

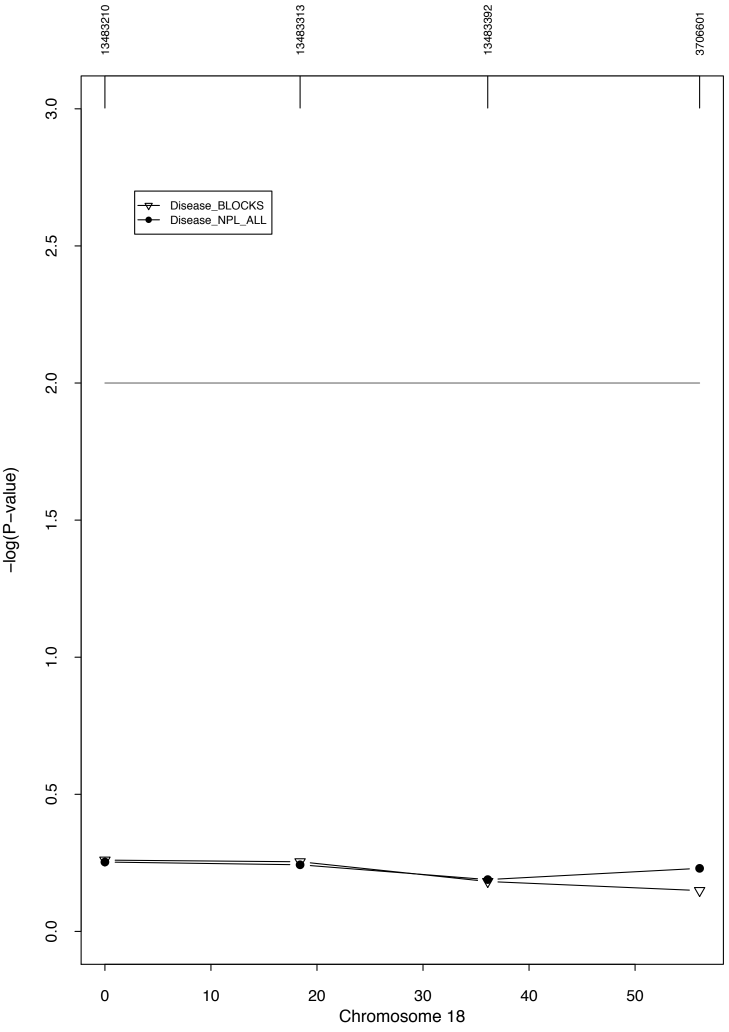
13483210
13483313
13483392
3706601
Disease_BLOCKS
Disease_NPL_ALL
−log(P−value)
Chromosome 18

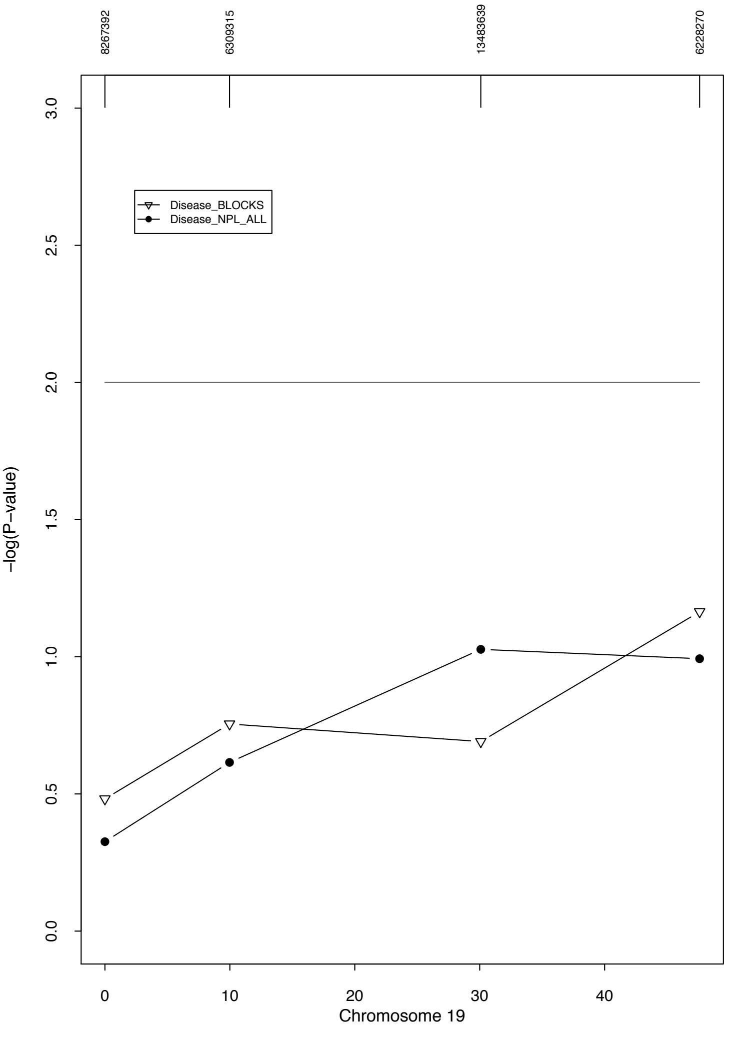
8267392
6309315
13483639
6228270
Disease_BLOCKS
Disease_NPL_ALL
−log(P−value)
Chromosome 19

**Supplemental Figure 3**

**Genome-wide linkage analysis of CHD at E9.5**

The *X*-axis represents the relative location of markers on a chromosome and the *Y*-axis represents the –log(p) for NPL-ALL scores and BLOCK recessive scores.

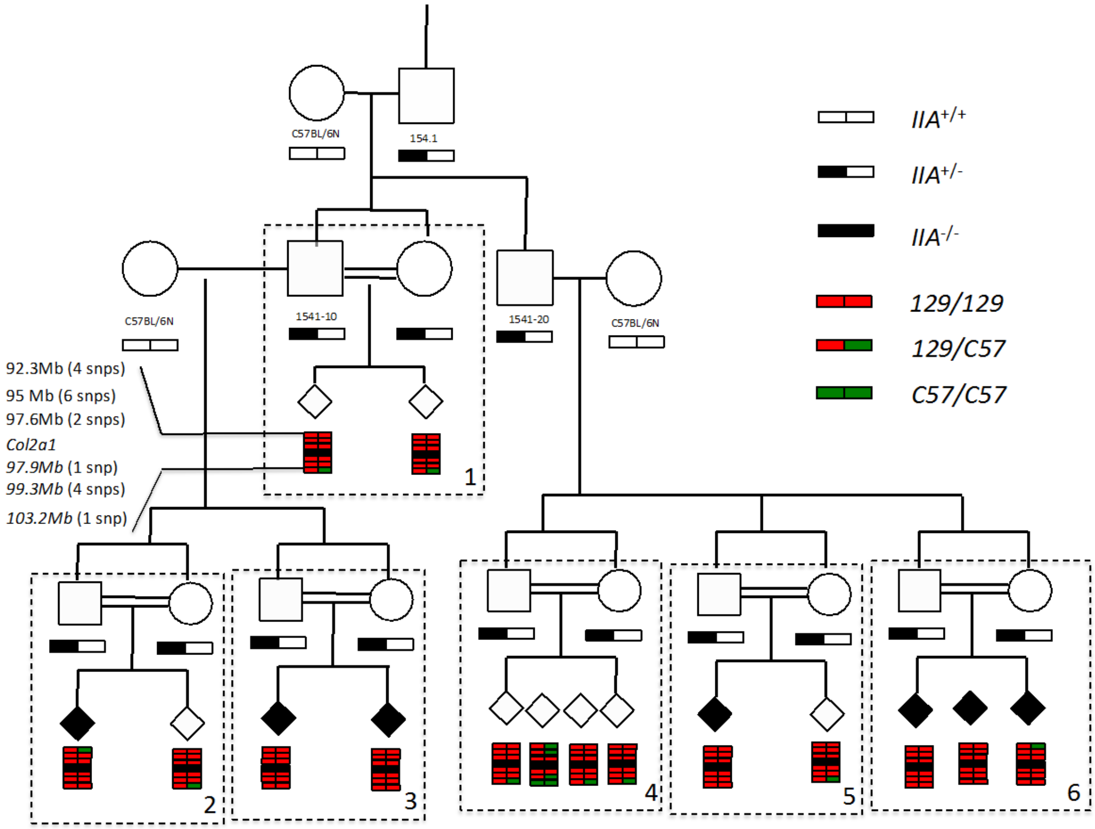


**Supplemental Figure 4**

**Haplotype configuration of a set of C57-*IIA*$^{-/-}$ mice on chr15:90.7-103.2Mb**

The figure shows a pedigree of a set of C57-*IIA*$^{-/-}$ with incomplete penetrance. 18 SNPs were genotyped for all the C57-*IIA*$^{-/-}$ mice. 129S1/SvImJ or 129SvEv allele is indicated with a red block. C57BL/6J or C57BL/6N is indicated with a green block. All SNPs, except rs6284372, had been previously tested to be able to distinguish 129SvEv from C57BL/6J. SNPs with the same genotype configurations would be grouped into one block. Positions shown on the left are the mean position of SNPs being grouped into one block.

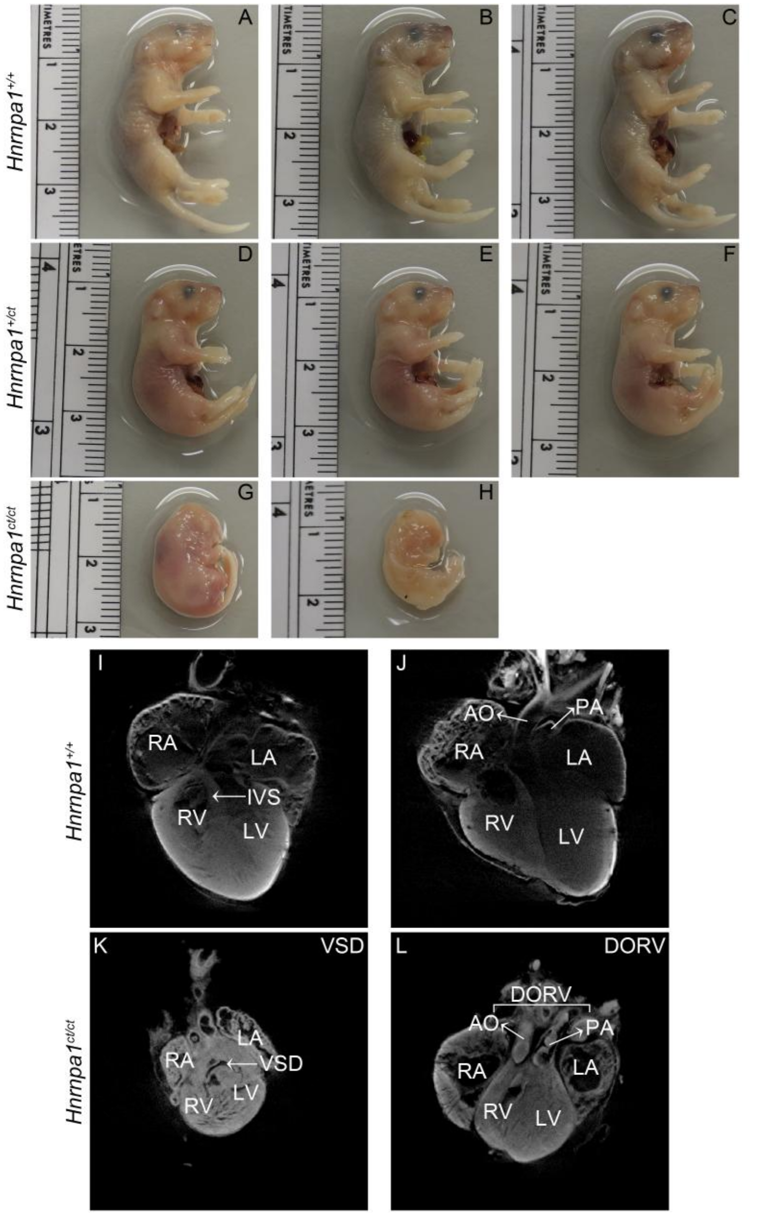


**Supplemental Figure 5**

**Cardiac defects in *Hnrnpa1*$^{ct/ct}$ P0 newborns**

Wild-type **(A-C)**, *Hnrnpa1*$^{+/ct}$ **(D-F)** and *Hnrnpa1*$^{ct/ct}$ **(G-H)** P0 newborns are shown in right lateral view. Cardiac defects in *Hnrnpa1*$^{ct/ct}$ P0 newborns: **(I)** Intact interventricular septum in the hearts of wild-type littermate controls. **(J)** The connection between the left ventricle and the aorta, as well as the normal spiralling relationship between the aorta and the pulmonary artery can be observed in the wild-type P0 hearts. **(K)** VSD is shown in one *Hnrnpa1*$^{ct/ct}$ mutant P0 heart. **(L)** DORV is observed in another *Hnrnpa1*$^{ct/ct}$ mutant P0 heart. Both the aorta and the pulmonary artery connect to the right ventricle. RA, right atrium; LA, left atrium; RV, right ventricle; LV, left ventricle; IVS, interventricular septum; AO, aorta; PA, pulmonary artery; VSD, ventricular septal defect; DORV, double outlet right ventricle.

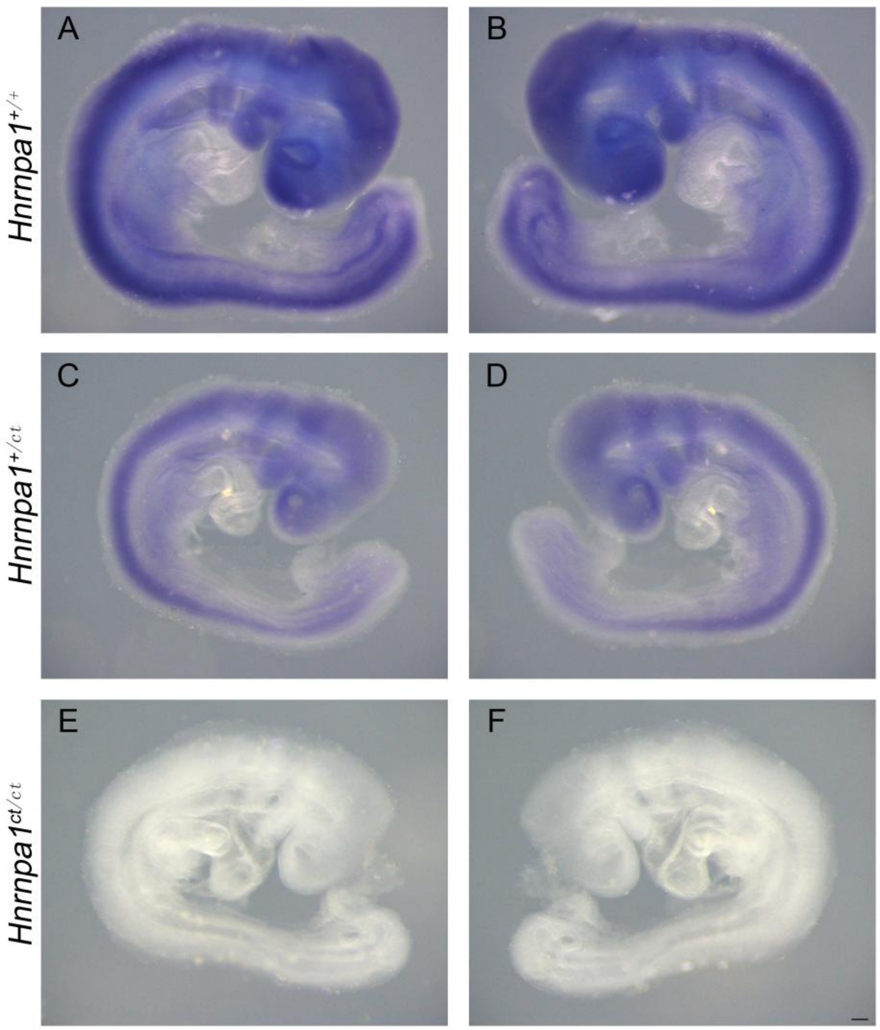


**Supplemental Figure 6**

**Whole mount *in situ* hybridization analyses of *Hnrnpa1* in wild-type littermate controls, *Hnrnpa1*$^{+/ct}$ heterozygous and *Hnrnpa1*$^{ct/ct}$ homozygous mutants at E9.5**

Embryos are shown in left and right lateral views. **(A-B)** *Hnrnpa1* mRNA is widely and strongly expressed in wild-type littermate controls. **(C-D)** Reduced expression level of *Hnrnpa1* mRNA is observed in *Hnrnpa1*$^{+/ct}$ heterozygous mutants. **(E-F)** Expression of *Hnrnpa1* mRNA is not detected in *Hnrnpa1*$^{ct/ct}$ homozygous mutants. Scale bar, 100μm.

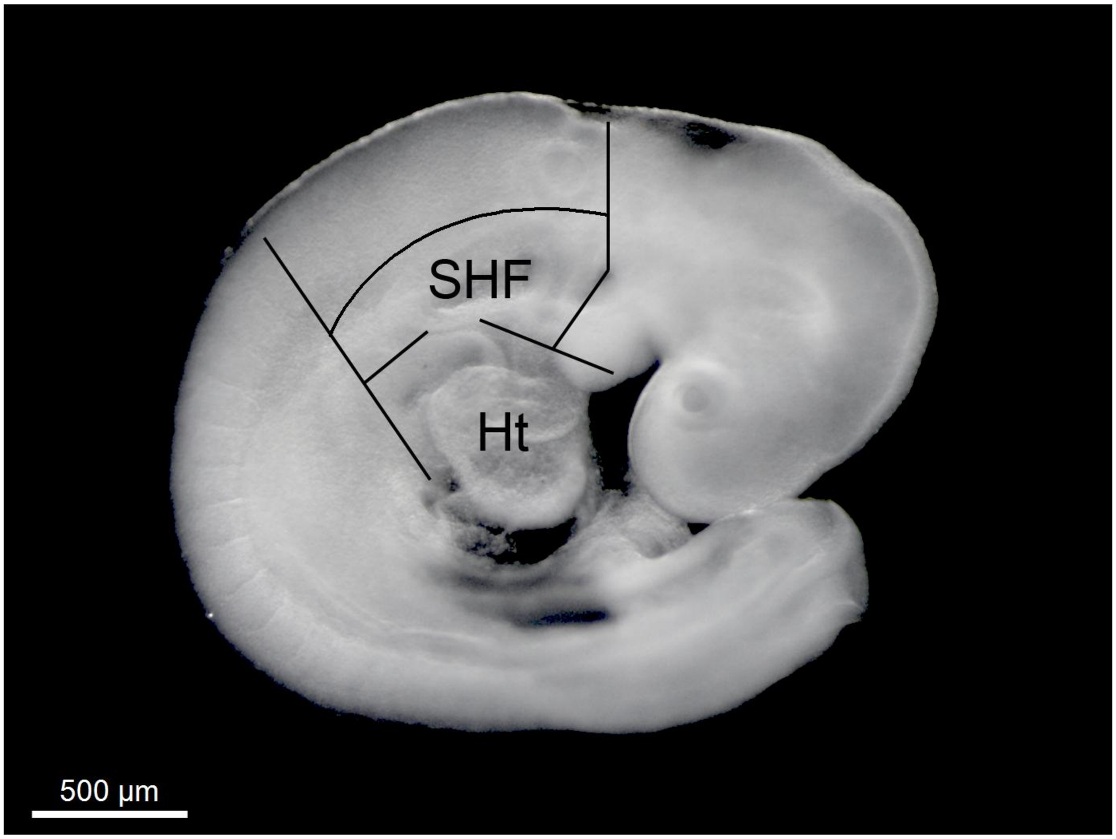


**Supplemental Figure 7**
**Isolation of the SHF and heart tube (Ht)**
Pharyngeal region and heart tube were isolated at E9.5 as shown in the photo.

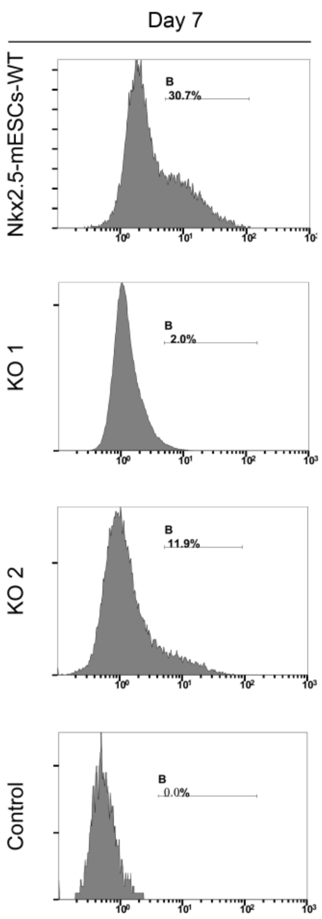


**Supplemental Figure 8**

**Percentage of Nkx2.5-EGFP positive cells was analyzed by flow cytometry at day 7**

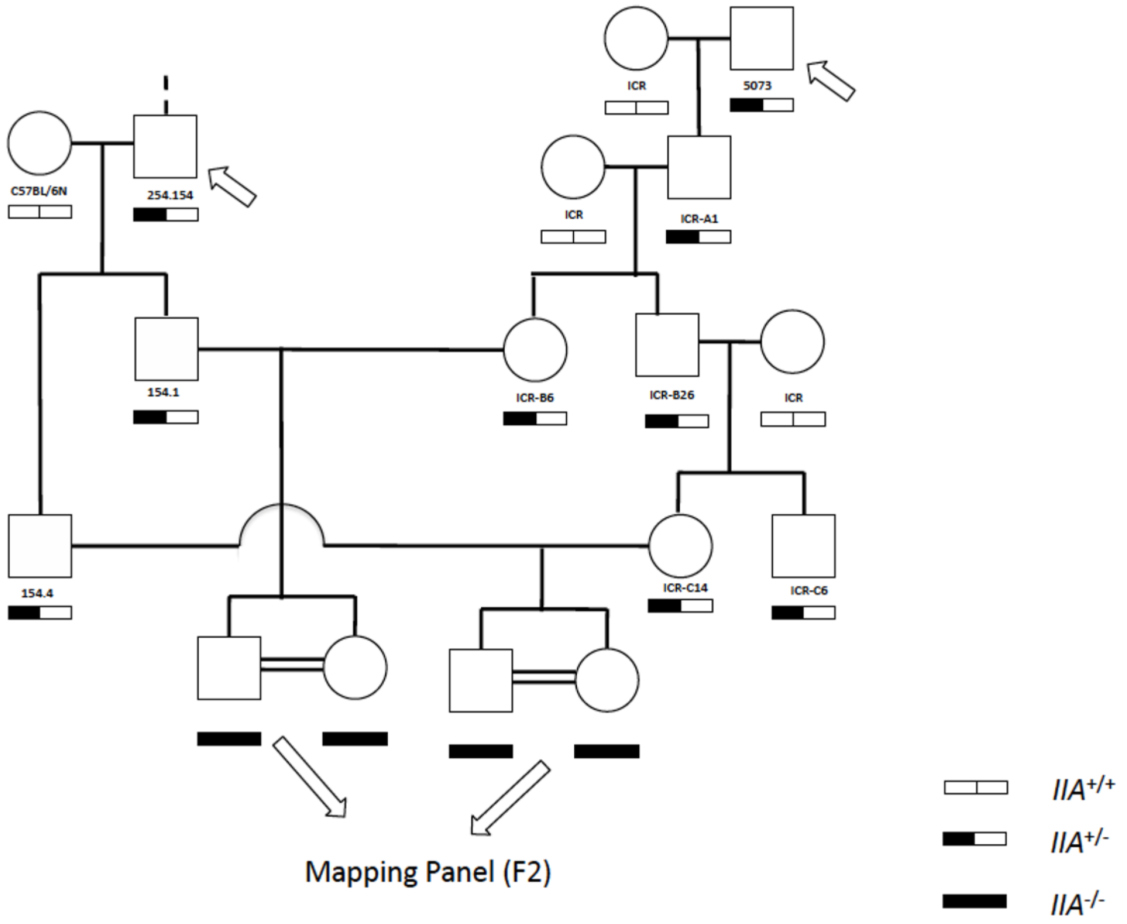


**Supplemental Figure 9**

**A mapping panel for a genome-wide linkage analysis**

A mapping panel for a genome-wide linkage analysis was generated by using F2 mice from C57-*IIA*$^{+/-}$ x ICR-*IIA*$^{+/-}$ crosses.

| | Mouse litter ID | | Normal | | Dead at birth |
|---|---|---|---|---|---|
| | | # backcross | % $IIA^{+/+}$ | % $IIA^{+/-}$ | % $IIA^{-/-}$ |
| Line 1 (C57BL/6N) | 3.9:A | >7 | 32.81 | 54.55 | 12.65** |
| Line 2 (129/SvEv) | 3.10:A | >15 | 20.63 | 55.56 | 23.81 |
| Line 3 (ICR) | 3.8:A | 2 | 24.53 | 49.06 | 26.42 |
| | 3.8:B | 4 | 37.84 | 40.54 | 21.62 |
| | 3.8:C | 6 | 18.42 | 60.53 | 21.05 |
| | 3.8:D | 7 | 23.08 | 47.44 | 29.49 |
| | 3.8: A+B+C+D | >1 | 24.67 | 49.33 | 26.00 |

**Supplemental Table 1**

**Recessive inheritance model of lethal in the line C57BL/6N background**

Three congenic lines were established by backcrossing to three inbred lines separately. The proportion of dead mice with $IIA^{-/-}$ genotypes was significantly deviated than that expected ($p = 1.2 \times 10^{-5}$) in the line with C57BL/6N background.

| dbSNP | Physical position (bp) |
|---|---|
| rs3717268 | 90759412 |
| rs13482709 | 91504598 |
| rs13482710 | 91723348 |
| rs3722990 | 91932927 |
| rs4137261 | 93751291 |
| rs3685284 | 94018477 |
| rs3657232 | 94145735 |
| rs13482721 | 94470493 |
| rs13482722 | 94682662 |
| rs13482723 | 94999572 |
| rs6285067 | 95144976 |
| rs13482726 | 95780106 |
| rs6208415 | 97720919 |
| rs13482732 | 97961248 |
| rs3708604 | 98955890 |
| rs6284372 | 99341295 |
| rs3703836 | 100416093 |
| rs3023430 | 100628798 |

**Supplemental Table 2**
**Selected markers for haplotype analysis**
These markers, except rs6284372, were all tested to be able to distinguish C57BL/6J from 129SvEv in our stock. rs6284372, however, can distinguish ICR from C57BL/6J or 129SvEv in our stock.

| CHD Phenotypes | Chinese patients | Pakistani patients |
|---|---|---|
| Atrial Septal Defects (ASD) | 43 | 0 |
| Ventricular Septal Defects (VSD) | 181 | 0 |
| Patent Ductus Arteriosus (PDA) | 29 | 50 |
| Tetralogy of Fallot (TOF) | 16 | 149 |
| Transposition of the great Arteries (TGA) | 1 | 26 |
| Pulmonary stenosis (PS) | 3 | 0 |
| Case total | 273 | 225 |
| Normal control | 300 | 0 |

**Supplemental Table 3**
**Human CHD patients from Chinese and Pakistani for screening of *HNRNPA1* gene**

| Primer ID | Sequence | Length of amplification fragment | Exon |
|---|---|---|---|
| E1-F | 5'CGAAGGTAGGCTGGCAGAT 3' | 333 | Exon 1 |
| E1-R | 5'AGTAACGAGTCCCGCATGAT 3' | | |
| E2-F | 5'GCGACCTGAACGAACAATAAG 3' | 295 | Exon 2 |
| E2-R | 5'CTGCACCAAGATAAGCCAAG 3' | | |
| E3-F | 5'CTTGGCTTATCTTGGTGCAG 3' | 470 | Exon 3 |
| E3-R | 5'CGAATCTTAAGTCCATAGCAGC 3' | | |
| E456-F | 5'GCTGCTATGGACTTAAGATTCG 3' | 823 | Exon 4-6 |
| E456-R | 5'CACCAAAGCCACCTGTAAG 3' | | |
| E7-F | 5'GGAGGAAACTTCAGTGGTCGT 3' | 382 | Exon 7 |
| E7-R | 5'TGCAAGTGTAACGGCTGAAG 3' | | |
| E8-F | 5'CTTCAGCCGTTACACTTGC 3' | 529 | Exon 8 |
| E8-R | 5'GCCACAGCAAGCAGAAGTATC 3' | | |
| E9-F | 5'GGTATGCTTGTGCCACTCTG 3' | 743 | Exon 9 |
| E9-R | 5'CTGCCACTGCCATAGCTACTG 3' | | |
| E10-F | 5'TCATGGGACCTCTTTACC 3' | 481 | Exon 10 |
| E10-R | 5'AGCTTCCCTGTCACTTCT 3' | | |

**Supplemental Table 4**
**Primers for sequencing of human *HNRNPA1***

| Genotyping | Primer ID | Sequence |
| --- | --- | --- |
| *IIA* | 5'Exon2 | 5'-TGTATGGAAGCCCTCATCTTG-3' |
| | mcol2intron1 | 5'-CCACCATTCCCTAGCATTTG-3' |
| | OYY-KO3 | 5'-TCATCCTTTCAACTCCCAGA-3' |
| rs32183020 | rs32183020-F | 5'-TCTCCTCCAGGAAAAGAGCA-3' |
| | rs32183020-R | 5'-AGAAAGCCCAGATTGGAGGT-3' |
| *Hnrnpa1* | *Hnrnpa1*-exon5-F | 5'-CCACCACACCTTCCCAGTC-3' |
| | *Hnrnpa1*-exon5-R | 5'-TTTTGGATCATTAGGCTAACCC-3' |

**Supplemental Table 5**
**Primers for genotyping**

| Primer ID | Sequence |
|---|---|
| *GAPDH*-Q-F | 5'-TGACGTGCCGCCTGGAGAAA-3' |
| *GAPDH*-Q-R | 5'-AGTGTAGCCCAAGATGCCCTTCAG-3' |
| *Hnrnpa1*-Q-F | 5'-ATTTTGAGCAGTATGGGAAG-3' |
| *Hnrnpa1*-Q-R | 5'-CGACCACCACCAAAGTTT-3' |
| *Acvr1*-Q-F | 5'- GCTGCATAGCAGATTTGGGC-3' |
| *Acvr1*-Q-R | 5'- CACTTCCGGAGCCATGTAGC-3' |
| *Bmpr1a*-Q-F | 5'- GGTTGTGCTCATTTCCATGGCT-3' |
| *Bmpr1a*-Q-R | 5'- TTGTAACGACCCCTGCTTGAGA-3' |
| *Fgf8*-Q-F | 5'-TGGAAGCAGAGTCCGAGTTC-3' |
| *Fgf8*-Q-R | 5'-TGTGAATACGCAGTCCTTGC-3' |
| *Fgf10*-Q-F | 5'-CAGTAAGACACGCAAGCATTTACTG-3' |
| *Fgf10*-Q-R | 5'-AATCTGATCCAATTCTTCCATGGT-3' |
| *Jag1*-Q-F | 5'- TGCTCACACCTGAAAGACCACT-3' |
| *Jag1*-Q-R | 5'- ATGGGGACCACAGACGTTAGAA-3' |
| *Isl1*-Q-F | 5'-CTGCTTTTCAGCAACTGGTCA-3' |
| *Isl1*-Q-R | 5'-TAGGACTGGCTACCATGCTGT-3' |
| *Mef2c*-Q-F | 5'-CCAGTGTCCAGCCATAACAGTTTG-3' |
| *Mef2c*-Q-R | 5'-AGATTCATAGGGGGAGGAGATTTG-3' |
| *Mlc2a*-Q-F | 5'-GGCACAACGTGGCTCTTCTAA-3' |
| *Mlc2a*-Q-R | 5'-TGCAGATGATCCCATCCCTGT-3' |
| *Mlc2v*-Q-F | 5'-ATCGACAAGAATGACCTAAGGGA-3' |
| *Mlc2v*-Q-R | 5'-ATTTTTCACGTTCACTCGTCCT-3' |
| *Myocd*-Q-F | 5'- GATGGGCTCTCTCCAGATCAG-3' |
| *Myocd*-Q-R | 5'- GGCTGCATCATTCTTGTCACTT-3' |
| *Nkx2.5*-Q-F | 5'-GCCGCCCCCACATTTTAC-3' |
| *Nkx2.5*-Q-R | 5'-CCATCCGTCTCGGCTTTGT-3' |
| *SRF*-Q-F | 5'-CTGCCTCAACTCGCCAGAC-3' |
| *SRF*-Q-R | 5'-TCAGATTCCGACACCTGGTAG-3' |
| *Tbx1*-Q-F | 5'-ACCGGTATGCTTTCCATAGCTCCT-3' |
| *Tbx1*-Q-R | 5'-AATCTGTTTCATCCACTGTGCGCC-3' |

**Supplemental Table 6**
**Primers for qRT-PCR**

| Genotyping | Primer ID | Sequence |
|---|---|---|
| Target primers | t1F | 5'-GGCCACAACTGTGAAGTAAGAAAG-3' |
| | t1R | 5'-CCAAAGCCACCTGGAAGAAAC-3' |
| Off-target primers | ot1F | 5'-CCAGGCTCTCTGTGGAGGTATG-3' |
| | ot1R | 5'-CTATGGTGGCAGGATCAAATGT-3' |
| | ot2F | 5'-TTATCCCCCTGTATGGAATGTC-3' |
| | ot2R | 5'-ATTTTGGGCTGCTCCTCGC-3' |
| | ot3F | 5'-GGAAGAAGCCAAGGCACCA-3' |
| | ot3R | 5'-GACGGTTTCACTCTTACCCTCTG-3' |

**Supplemental Table 7**
**Primers for CRISPR knockout of *Hnrnpa1* in mESCs**
